# A comparison of intestinal microbiota in a population of low-risk infants exposed and not exposed to intrapartum antibiotics: The Baby & Microbiota of the Intestine cohort study protocol

**DOI:** 10.1186/s12887-016-0724-5

**Published:** 2016-11-10

**Authors:** Julia Simioni, Eileen K. Hutton, Elizabeth Gunn, Alison C. Holloway, Jennifer C. Stearns, Helen McDonald, Andrea Mousseau, Jonathan D. Schertzer, Elyanne M. Ratcliffe, Lehana Thabane, Michael G. Surette, Katherine M. Morrison

**Affiliations:** 1 Midwifery Education Program, McMaster University, Hamilton, ON Canada; 2 Department of Obstetrics and Gynecology, McMaster University, Hamilton, ON Canada; 3 Department of Pediatrics, McMaster University, HSC 3A59 1280 Main St W, L8N 3Z5 Hamilton, ON Canada; 4 Department of Medicine, McMaster University, Hamilton, ON Canada; 5 Department of Biochemistry and Biomedical Sciences, McMaster University, Hamilton, ON Canada; 6 Farncombe Family Digestive Health Research Institute, McMaster University, Hamilton, ON Canada; 7 Department of Clinical Epidemiology and Biostatistics, McMaster University, Hamilton, ON Canada; 8 Centre for Evaluation of Medicines, St. Joseph’s Healthcare Hamilton, Hamilton, ON Canada

**Keywords:** Microbiome, Infant, Cohort study, Antibiotics, Birth

## Abstract

**Background:**

The intestinal microbiota influences metabolic, nutritional, and immunologic processes and has been associated with a broad range of adverse health outcomes including asthma, obesity and Type 2 diabetes. Early life exposures may alter the course of gut microbial colonization leading to differences in metabolic and immune regulation throughout life. Although approximately 50 % of low-risk full-term infants born in Canada are exposed to intrapartum antibiotics, little is known about the influence of this common prophylactic treatment on the developing neonatal intestinal microbiota. The purpose of this study is to describe the intestinal microbiome over the first 3 years of life among healthy, breastfed infants born to women with low-risk pregnancies at full term gestation and to determine if at 1 year of age, the intestinal microbiome of infants exposed to intrapartum antibiotics differs in type and quantity from the infants that are not exposed.

**Methods:**

A prospectively followed cohort of 240 mother-infant pairs will be formed by enrolling eligible pregnant women from midwifery practices in the City of Hamilton and surrounding area in Ontario, Canada. Participants will be followed until the age of 3 years. Women are eligible to participate in the study if they are considered to be low-risk, planning a vaginal birth and able to communicate in English. Women are excluded if they have a multiple pregnancy or a preterm birth. Study questionnaires are completed, anthropometric measures are taken and biological samples are acquired including eight infant stool samples between 3 days and 3 years of age.

**Discussion:**

Our experience to date indicates that midwifery practices and clients are keen to participate in this research. The midwifery client population is likely to have high rates of breastfeeding and low rates of intervention, allowing us to examine the comparative development of the microbiome in a relatively healthy and homogenous population. Results from this study will make an important contribution to the growing understanding of the patterns of intestinal microbiome colonization in the early years of life and may have implications for best practices to support the establishment of the microbiome at birth.

**Electronic supplementary material:**

The online version of this article (doi:10.1186/s12887-016-0724-5) contains supplementary material, which is available to authorized users.

## Background

The intestinal microbiota is essential to metabolic, nutritional and immunologic processes. The microbiome of healthy adults varies significantly between individuals. Differences in diversity or relative abundance of microbes has been associated with a broad range of adverse health outcomes including: obesity, inflammatory bowel disease, atopic disease, Type 2 diabetes, [1] multiple sclerosis, [2] mental health or behaviour disorders, including autism [3] and alcoholism [4]. The adult microbiota can be transiently altered by diet, infection and antibiotic use, but in most individuals it reverts to the composition observed before the perturbation [5–8].

In the newborn infant, following birth, colonisation of the gut typically proceeds rapidly in the first hours and days following initial exposure to maternal vaginal and rectal microbiota and subsequently to maternal skin and breast milk [9, 10]. Compared to adults, the infant microbiome is quite unstable, has greater inter- and intra-individual variation, has a lower number of species and has a higher proportion of *Bifidobacterium* [11]. Microbial communities in the young child stabilize over time and begin to resemble those in adults between 1 and 3 years of age [12]. Factors such as the timing of weaning from breast milk as well as the type and timing of the introduction of solid foods may contribute to this process, [13, 14] but require further exploration. Membership of microbial communities and colonization patterns has been shown to differ by mode of birth. Caesarean section and exposure of the newborn to antibiotics following birth cause deviation from normal colonization patterns with disturbances in and poor diversity of intestinal microbiota and these differences have been shown to persist for up to 1 year [15–18]. Caesarean section has been associated with a number of long-term health outcomes and the gut microbiome has been implicated in the biological pathway [19, 20]. Thus, early exposure to the appropriate colonizing organisms is potentially critical to long-term health.

**Fig. 1:**
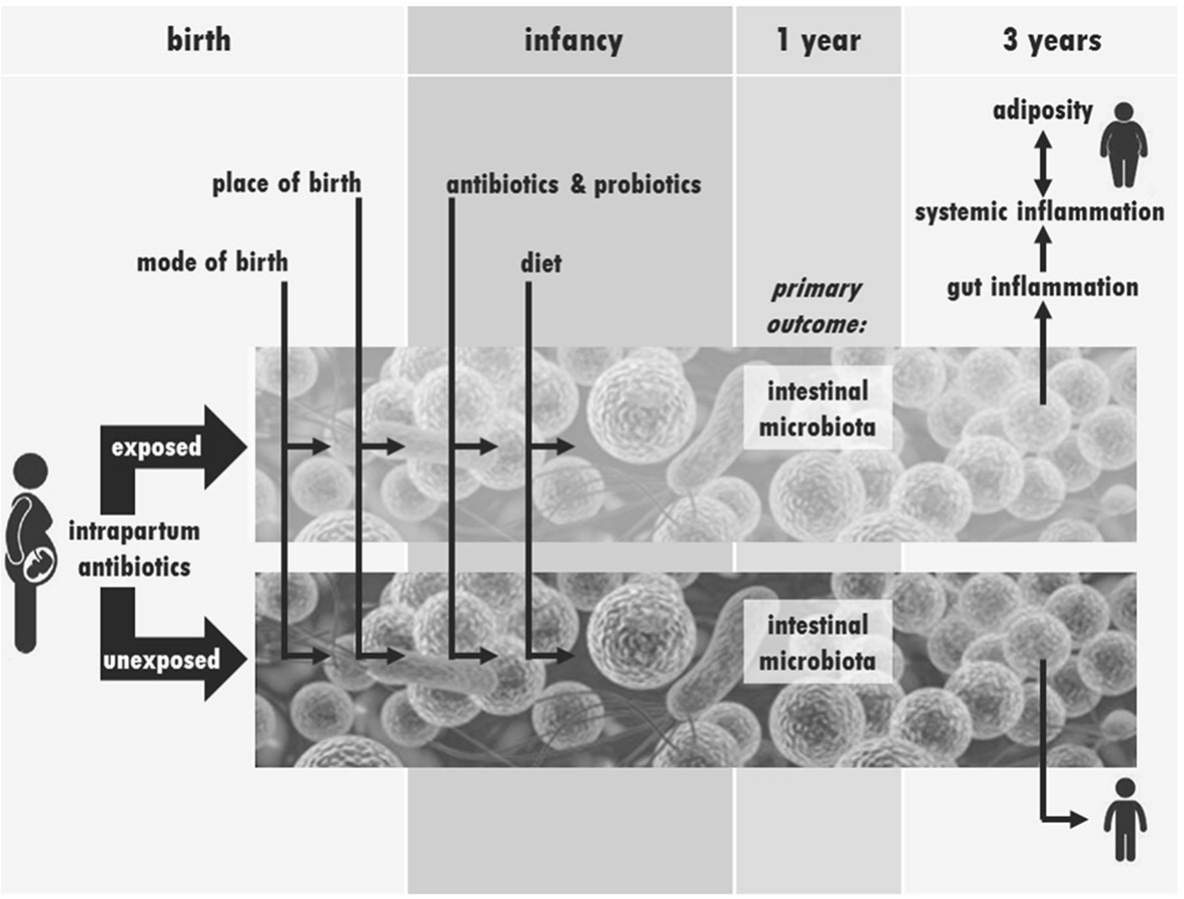
Baby & Mi Study Framework. The cohort of women-infant dyads will be classified as either being exposed to intrapartum antibiotics for *Group B Streptococcus* prophylaxis or unexposed. We will also collect information about other factors hypothesized to impact the intestinal microbiota at birth and during infancy/early childhood. The primary outcome is the intestinal microbiota at 1 year. Secondary outcomes include intestinal microbiota, adiposity and markers of gut and systemic inflammation at 3 years. The hypothesized relationships between these outcomes is shown using arrows

As many as 50 % of low-risk, full term infants born in Canada are exposed to intrapartum antibiotic prophylaxis (IAP); about 30 % because of prophylactic management for *Group B Streptococcus* (GBS) [21] and the remainder due to antibiotic coverage used for Caesarean birth. IAP has the potential to impact early development of neonatal intestinal microbiota, first because of the changed maternal vaginal and rectal microbiota to which the neonate is exposed during birth, and secondly due to the direct exposure of the fetus to the antibiotic. Therefore, the primary objective of our study is to determine if infants born to women who receive IAP for GBS or a similar regimen have intestinal microbiota at 1 year that differs significantly in type and quantity from those not exposed to IAP (Fig. 1).

## Methods/Design

### Study design and setting

A prospectively followed cohort of 240 mother-infant pairs will be formed by enrolling eligible pregnant women from midwifery practices in the City of Hamilton and surrounding area in Ontario, Canada. A pilot cohort (83 mother-infant pairs) was enrolled between July 1^st^ 2012 and December 31^st^ 2013. Recruitment for the second phase of the study began October 1^st^ 2014 and is ongoing. Participants will be followed until the age of 3 years. The study was approved by the joint Hamilton Health Sciences - McMaster University Research Ethics Board and by Research Ethics Boards at all participating healthcare organizations.

### Study population

Women are eligible to participate in the study if they are considered to be low-risk (defined as being under the care of a midwife), planning a vaginal birth and able to communicate in English in order to provide signed informed consent. Women are excluded from the study if they have a known multiple pregnancy or a preterm birth (before 37 completed weeks gestation). Women who are enrolled prior to 37 completed weeks gestation are contacted by telephone after 37 completed weeks to reassess their eligibility.

### Recruitment

The Baby & Mi study is advertised through pamphlets and posters in the waiting areas at eight participating midwifery practices and on social media. The midwives and administrative staff are asked to inform their pregnant clients about the study. ‘Consent to Contact’ forms are completed by mothers interested in hearing more about the study and research personnel then contact them by telephone or email. Study visits are arranged with women who agree to participate.

### Data collection

After providing informed consent, women are asked to complete a baseline questionnaire that collects information about their pregnancy and family medical history. Women enrolled at a gestational age less than 37 completed weeks are contacted by telephone within 3 weeks of their estimated due date to reassess time-dependent characteristics such as smoking status and medication use during pregnancy. Case report forms are available in Additional file 1.

Data about the pregnancy and birth are recorded onto paper case report forms from antenatal forms, the birth record and mother and infant charts by the midwives. On day 3, day 10 and 6 weeks postpartum participants are instructed to complete follow up questionnaires and to place a diaper containing stool in a labeled study bag and store the sample frozen in their household freezer until their study visit when the baby is 12 weeks of age. The women are reminded to complete these tasks on the appropriate days by research personnel through email or text message. In order to facilitate recall throughout the follow up period, mothers are given a study diary that provides them with a place to record events such as infections, medications and sleep disturbances.

Participants are asked to attend follow up visits at McMaster University Medical Centre at 12 weeks, 5 months, 1 year, 2 years and 3 years of age for stool sample delivery, follow up data collection and measurements of infant growth. Participants of the pilot study were also asked to attend a follow up visit at 6 weeks postpartum. Women are contacted between study visits to complete questionnaires online or by telephone. A summary of the data collection time points can be found in Table 1.

**Table 1:** Data Collection Timeline

Timepoint	Data collection					
Baseline		Self-completed Questionnaire				
36 to 37 weeks’ gestation		Telephone questionnaire				
Birth		Midwife-completed questionnaire				
Day 3		Self-completed Questionnaire	Stool sample			
Day 10		Self-completed Questionnaire	Stool sample			
6 weeks		Self-completed Questionnaire	Stool sample			
12 weeks	Study visit	Self-completed Questionnaire	Stool sample	Anthropometric measurements	PEA POD	
4 months		Telephone questionnaire				
5 months	Study visit	Self-completed Questionnaire	Stool sample	Anthropometric measurements	PEA POD	
6 to 10 months		Telephone questionnaires				
1 year	Study visit	Self-completed Questionnaire	Stool sample	Anthropometric measurements		
2 years	Study visit	Self-completed Questionnaire	Stool sample	Anthropometric measurements		
3 years	Study visit	Self-completed Questionnaire	Stool sample	Anthropometric measurements	DXA	Blood draw

### Exposure assessment

Infants born to women who are administered prophylactic antibiotic treatment for GBS or a similar regimen in labour will be classified as IAP exposed. The unexposed group includes infants born to women who are not exposed to IAP. Women received IAP under the recommendations of their care providers according to standard care practices. Information about intrapartum antibiotic use is collected from the birth record including indication, dose, frequency and time to delivery from first dose.

### Outcome assessment

The primary outcome is the type and abundance of species present in the intestinal microbiota at 1 year of age, which will be determined through bacterial diversity analysis and microbiota profiling of stool specimens This comparison will also be made using stool samples collected at day 3 and 10, 6 and 12 weeks, 5 months, 2 and 3 years of age.

#### Gut microbiome measurement

DNA extraction from stool is carried out using a previously described protocol that enhances DNA recovery from microbial communities [22, 23] with modifications to increase quantitative recovery of bacteria across different taxa [24]. Upon delivery to the laboratory, stool samples are thawed and DNA is extracted. Approximately 100 mg of stool is sampled from each diaper. Where the stool is fully absorbed into the diaper, a 1 cm by 1 cm square of diaper is cut using sterile scissors and used for DNA extraction. Bacterial community profiling of the 16S rRNA gene is carried out using paired end reads of the V3 region using barcoded Illumina sequencing as described previously [25]. Sample preparation and 250 paired-end sequencing is carried out on a MiSeq Illumina sequencer as per manufacturer’s instructions. This provides approximately 50,000–100,000 reads per sample that is processed by an in-house bioinformatics pipeline [24] and the output includes clustered sequences in operational taxonomic units (OTUs) and taxonomic assignments as described previously [26, 27].

Growth is monitored at each follow-up visit using standard approaches for the measurements of length, weight (including weight to length ratio and peak weight velocity), skinfold thickness (tricep, subscapular, bicep and suprailiac), head and hip circumference. The rate of fat accretion will be determined using measurements of body composition at 6 weeks (pilot participants only), 12 weeks, 5 months and 3 years. At 6 weeks, 12 weeks and 5 months of age an air displacement plethysmography system (PEA POD) is used to measure percent fat. At 3 years of age body composition is measured using dual-energy X-ray absorptiometry (DXA).

Atopic disease is assessed during study visits and telephone contacts by asking mothers if they suspect or have been told by a physician that their child has eczema, asthma, reactive airway disease or allergies. Symptoms of eczema are also assessed using the Sampson Oranje criteria for eczema [28] and the core questionnaire for eczema that was developed by the International Study of Asthma and Allergies in Childhood [29]. Sleep patterns are reported at each contact using questions from the Brief Index Sleep Questionnaire [30]. Symptomatic gastroesophageal reflux is evaluated at 6 weeks, 12 weeks, 5 months and 1 year using the Infant Gastroesophageal Reflux Questionnaire Revised [31]. Gastrointestinal problems are measured at 30 months using the PedsQL-Gastrointestinal Symptoms Scale [32]. Measures of cardiometabolic health and systemic inflammation will be made using fasting blood collected from each participant at 3 years of age. This will include lipids, glucose and inflammatory markers. Fecal calprotectin will be measured at 3 years of age as a marker for gut inflammation. Child behaviour and temperament will be measured using the Strengths and Difficulties [33] and the Children’s Behaviour Very Short Form questionnaires [34].

### Covariate assessment

Additional information is collected to describe the study population. Several variables will be assessed as confounders or effect modifiers of the relationship between IAP exposure and intestinal microbiota colonisation and secondary outcomes. Covariates that are collected at baseline include: maternal age, maternal pre-pregnancy weight, maternal and household smoking status, medications taken during pregnancy and first degree relative history of atopic disease, obesity, allergies or heart disease. Information about the pregnancy and birth are collected from birth records and charts including: GBS screening results, mode of delivery, place of birth (home or hospital), birth weight, length at birth, sex of the newborn, Apgar scores, maternal and infant antibiotic use prior to hospital discharge, intensive care unit admission and time from birth until discharge or the midwife leaving (homebirth). At follow up visits and through telephone contacts, parents are asked about time-varying covariates such as maternal and child exposure to antibiotics or antifungals, other medication or health product use, vaccine history, travel history, pets, daycare, exposure to tobacco smoke and changes in feeding (breast, formula or combination) including the addition of solid food.

### Sample size

Developing a sample size for studies in which the microbiota is the primary outcome is challenging. To date, most studies investigating the infant microbiome have included less than 100 subjects. To assess the feasibility of our study design, a sample size of 80 mother-infant pairs was selected. Recruitment and the collection of stool and data was found to be feasible, and attrition was low (6 %). Therefore we determined that enrolling 160 mother-infant pairs in Phase 2, for a total sample size of 240, was achievable.

### Statistical analyses

We will use a diagram to summarize the patient flow in the study. Demographic and prognostic baseline characteristics will be reported as mean (standard deviation) for continuous variables and count (percent) for categorical variables. We will use regression analysis to analyze data for all clinical outcomes with intrapartum exposure to antibiotics as an independent variable after adjusting for confounders. Clinically important covariates will be investigated as potential confounders where appropriate for each outcome. The results will be expressed as odds ratio [OR] (for logistic regression for binary outcomes) or coefficient (for linear regression for continuous outcomes), corresponding standard error, 95 % confidence intervals and associated *p*-values. *P*-values will be reported to three decimal places with *p*-values less than 0.001 reported as *p* < 0.001. For all tests, we will use alpha = 0.05 level of significance. Assessment of model assumptions for regression analyses will be done by examining the residuals.

There is likely to be missing data that will likely increase with duration of the follow-up. We will use multiple imputation [35] to handle missing data. We will use generalized estimating equations (GEE) [36] to account for possible serial correlation of measurements within a participant overtime. Unlike ordinary regression analysis, GEE allows accounting for the possible correlation of outcomes for participants over time. Lastly, we will use propensity score methods to address the differential propensity for infant antibiotic exposure. All analyses will be performed using R or SPSS statistical software.

To understand the influence of IAP on the infant gut microbiota, we will analyze the microbial communities using culture-independent microbiota profiling methods. Microbiome analysis will include α-diversity metrics for each sample and β-diversity measures (Bray-Curtis) and other statistical analysis in R [37]. Association of microbial community differences with sample groups will be assessed with permutational multivariate analysis of variance using Bray-Curtis dissimlarities (with vegan package in R) [38]. Association of taxon abundance with sample groups will be assessed with a generalized linear mixed model with age and intrapartum exposure as fixed effects and individual child as a random effect using the lme4 package in R [39]. Functional properties of the microbiota will be inferred using PICRUSt (Phylogenetic Investigation of Communities by Reconstruction of Unobserved States) [40].

## Discussion

### Feasibility

We enrolled 32 % of women who completed a ‘Consent to Contact’ form during the recruitment phase of our pilot study. The rates of follow-up and of data completion have been excellent. Of the first 83 participants recruited, one participant became ineligible prior to delivery (high risk pregnancy identified). Two participants have withdrawn; one prior to delivery and one at 12 weeks postpartum. Two participants were lost to follow up, resulting in 78 (94 %) participants remaining in the pilot cohort. In the pilot cohort, 94 % of follow up visits up to and including the 1-year visit were attended.

### Strengths & limitations

We are developing a cohort of mother-infant pairs derived from the midwifery client population, providing us with the opportunity to examine a healthy pregnant and newborn population with low intervention rates and high breast-feeding rates. The homogeneity of this population may allow us to better examine relationships between the microbiome and exposures of interest. The midwifery population also gives us the opportunity for novel observations including place of birth (home and hospital) as a potential covariate. However, our selection of a low-risk cohort will preclude evaluation of nutritional intake that does not include breastfeeding or of exposures that occur with higher risk pregnancies or following preterm birth. Thus, our findings will need to be verified in these populations.

The investigative team is inter-professional in nature, has complementary areas of expertise, and includes national leaders in their respective fields. Our team provides expertise in pediatrics, obstetrics, midwifery, biostatistics, epidemiology, basic science including animal modeling, and microbiology, and is well suited both to undertake this research and to bring results back to the clinical practice arenas where the findings will be most relevant.

### Implications

Our research will provide documentation of the development of the gut microbiome in a population of healthy full term infants born to low-risk women at full term gestation. By comparing the outcomes of exposed and unexposed infants we provide a first step in evaluating potential unforeseen negative consequences later in life of the current GBS clinical guidelines advocating IAP for GBS colonized women. Our team will explore this complex issue with examination of outcomes at both the microbiological level by examining intestinal colonisation in the first 3 years of life and in exploring the influence on systemic inflammation, adiposity, atopic disease and other outcomes of interest. Results from this study will make an important contribution to the growing body of knowledge and understanding of the patterns of intestinal microbiome establishment in the early years of life and the impact of IAP on the development of intestinal microbiota in newborns.

## Additional file

Additional file 1: Case Report Forms. This file contains the case report forms to be completed by participants and research staff from the time of eligibility assessment and enrollment to the time of the 3 year study visit. (PDF 1007 kb)

## Abbreviations

DXA: Dual-energy X-ray absorptiometry
GBS: *Group B Streptococcus*
GEE: Generalized estimating equations
IAP: Intrapartum antibiotic prophylaxis
OTU: Operational taxonomic units
PICRUSt: Phylogenetic investigation of communities by reconstruction of unobserved states
