# Supplementary material for: A comparison of intestinal microbiota in a population of low-risk infants exposed and not exposed to intrapartum antibiotics: The Baby & Microbiota of the Intestine cohort study protocol
Source: BMC Pediatr. 2016 Nov 10;16:183. doi: 10.1186/s12887-016-0724-5 (PMC5103394; doi:10.1186/s12887-016-0724-5)
Supplement: Additional file 1: — Case Report Forms. This file contains the case report forms to be completed by participants and research staff from the time of eligibility assessment and enrollment to the time of the 3 year study visit. (PDF 1007 kb) [file 12887_2016_724_MOESM1_ESM.pdf]

Baby & Mi

ELIGIBILITY ASSESSMENT

Instructions:
- **This form is to be completed by research personnel prior to enrolment.**

Date of eligibility assessment: ☐☐☐☐ / ☐☐ / ☐☐
year month day

1. Are you planning to have a vaginal birth?
   - ☐ No → **ineligible**
   - ☐ Yes

2. Are you having a single baby (not twins or triplets)?
   - ☐ No → **ineligible**
   - ☐ Yes

3. Are you able to communicate in English?
   - ☐ No → **ineligible**
   - ☐ Yes

Does this woman meet all study eligibility criteria by answering "Yes" to all of the questions above?
- ☐ Yes → **eligible to enroll**
- ☐ No

Did this woman sign their Patient Informed Consent form, including date and witness signature?
- ☐ Yes
- ☐ No → **do not enroll**

Person must first meet all study eligibility criteria and sign consent form, before receiving Participant ID (PID). Obtain PID from Study Participant Tracking Sheet.

Participant ID: ☐☐ – ☐☐☐

Baby & Mi

PID: ☐☐ – ☐☐☐

Mother's Date of Birth: ☐☐☐☐ ☐☐ ☐☐
year month day

BASELINE INFORMATION

Instructions:
- **This form is to be completed by study participants at enrolment.**
- **Mark only one box with an "X" for each question, unless instructed to do otherwise.**
- **If you do not understand a question, please ask a research staff member.**
- **All of your answers will remain confidential.**

1. What is today's date? ☐☐☐☐ / ☐☐ / ☐☐
   year month day

2. What is your expected date of birth? ☐☐☐☐ / ☐☐ / ☐☐
   year month day

3. What was your weight just before this pregnancy? ☐☐☐ lbs or ☐☐☐ kg

4. Have you been regularly exposed to other people's tobacco smoke during your pregnancy? (regularly is defined as most days or nights)
   - ☐ No
   - ☐ Yes
     - → if yes, ***not counting yourself***, how many people who live in your household smoke regularly?
       ☐☐ people
     - → if yes, do people smoke regularly in the room where you work?
       - ☐ No
       - ☐ Yes
     - → if yes, how many hours per day are you exposed to other people's tobacco smoke in total (at home, at work and elsewhere)?
       ☐☐ hours

5. Do you smoke cigarettes?
   - ☐ Never smoked
   - ☐ Smoked prior to pregnancy, but not now
   - ☐ Smoked earlier in pregnancy, but not now
   - ☐ Current smoker
     - → if you are a current smoker, how many cigarettes do you usually smoke per day?
       ☐☐ cigarettes per day

Baby & Mi

PID: ☐☐ – ☐☐☐

Mother's Date of Birth: ☐☐☐☐ ☐☐ ☐☐
year month day

BASELINE INFORMATION

---

6. Do you, your baby's father or your other child/children (if applicable) have any of the following: (check all that apply)

	YOURSELF			FATHER OF BABY			N/A, no other children ❑<br>YOUR CHILD/ CHILDREN		
	No	Yes	Unknown	No	Yes	Unknown	None	Yes, 1 or more children	Unknown
Asthma, wheezing	❑	❑	❑	❑	❑	❑	❑	❑	❑
Eczema	❑	❑	❑	❑	❑	❑	❑	❑	❑
Seasonal allergies	❑	❑	❑	❑	❑	❑	❑	❑	❑
Food allergies	❑	❑	❑	❑	❑	❑	❑	❑	❑
Other allergies	❑	❑	❑	❑	❑	❑	❑	❑	❑
Diabetes	❑	❑	❑	❑	❑	❑	❑	❑	❑
High blood pressure	❑	❑	❑	❑	❑	❑	❑	❑	❑
Heart disease (heart attack, angina, bypass surgery)	❑	❑	❑	❑	❑	❑	❑	❑	❑
Obesity or overweight	❑	❑	❑	❑	❑	❑	❑	❑	❑

7. Please list any medications, health products (including vitamins, probiotics, supplements and nicotine replacement therapies like "the patch"), alcohol or street drugs that you have taken during this pregnancy.

❑ None

Name	When during this pregnancy? (check both if it applies)<br>**Before 20 weeks**	**After 20 weeks**<br>❑ N/A, not yet 20 weeks
a. ______________________________	❑	❑
b. ______________________________	❑	❑
c. ______________________________	❑	❑
d. ______________________________	❑	❑
e. ______________________________	❑	❑
f. ______________________________	❑	❑

Baby & Mi

PID: ☐☐ – ☐☐☐

Mother's Date of Birth: ☐☐☐☐ ☐☐ ☐☐
year month day

BASELINE INFORMATION UPDATE

Instructions:
- **This form is to be completed by research personnel through telephone contact with participants.**
- **Refer to the participant's Baseline Form for Question #9.**
- **Participants who were less than 36 weeks, 0 days gestation at enrolment are to be contacted between 36 weeks, 0 days gestation and 36 weeks, 6 days gestation (inclusive).**

1. Was the baseline information update done?
   - ☐ No, because the participant was enrolled at or after 36 weeks, 0 days gestation
   - ☐ No, because the participant could not be contacted
   - ☐ Yes

2. Date of phone call: ☐☐☐☐ / ☐☐ / ☐☐
   year month day

3. Are you still pregnant?
   - ☐ No → **ineligible for further follow-up**
   - ☐ Yes

4. Are you still planning to have a vaginal birth?
   - ☐ No → **ineligible for further follow-up**
   - ☐ Yes

5. Are you having a single baby (not twins or triplets)?
   - ☐ No → **ineligible for further follow-up**
   - ☐ Yes

6. Is a midwife still your primary care provider?
   - ☐ No → **ineligible for further follow-up**
   - ☐ Yes

Script if they answer "no" to any of questions 3, 4, 5 or 6:
Researcher: "Ok that is all I need to know from you today. This study is going to continue to follow women after their births if they are not pre-term, planning a C-section, having multiples or high risk, so at this point, you have already provided us with as much information as we need. We really appreciate your participation in our study—thank you so much for your time. Do you have any questions for me?"

PID: ☐☐–☐☐☐

Mother's Date of Birth: ☐☐☐☐ ☐☐ ☐☐
year month day

BASELINE INFORMATION UPDATE

---

7. Have you been regularly exposed to other people's tobacco smoke during your pregnancy? (regularly is defined as most days or nights)
   - ❑ No
   - ❑ Yes
     - → if yes, ***not counting yourself***, how many people who live in your household smoke regularly?
       ☐☐ people
     - → if yes, do people smoke regularly in the room where you work?
       - ❑ No
       - ❑ Yes
     - → if yes, how many hours per day are you exposed to other people's tobacco smoke in total (at home, at work and elsewhere)?
       ☐☐ hours

8. Do you smoke cigarettes?
   - ❑ Never smoked
   - ❑ Smoked prior to pregnancy, but not now
   - ❑ Smoked earlier in pregnancy, but not now
   - ❑ Current smoker
     - → if you are a current smoker, how many cigarettes do you usually smoke per day?
       ☐☐ cigarettes per day

BASELINE INFORMATION UPDATE

9. Please list any medications, health products (including vitamins, probiotics, supplements and nicotine replacement therapies like "the patch"), alcohol or street drugs that you have taken during this pregnancy ***and not told us about yet***. **Note to interviewer:** refer to Baseline Form to review previously reported health products.

   ☐ None

Name	When during this pregnancy? (check both if it applies) Before 20 weeks	After 20 weeks
a. ______________________________	☐	☐
b. ______________________________	☐	☐
c. ______________________________	☐	☐
d. ______________________________	☐	☐
e. ______________________________	☐	☐
f. ______________________________	☐	☐

PID: ☐☐ – ☐☐☐

Mother's Date of Birth: ☐☐☐☐ ☐☐ ☐☐
year month day

BIRTH

Instructions:
- **This form is to be completed by the midwives using the birth record, nursing notes and newborn admission forms as needed.**

1. Maternal height: ☐ feet, ☐☐ inches or ☐☐☐ cm

   Date of last prenatal visit: ☐☐☐☐ / ☐☐ / ☐☐
   year month day

2. Maternal weight at last prenatal visit: ☐☐☐ lbs or ☐☐☐ . ☐ kg

3. Gravida: ☐☐

4. Term: ☐☐

5. Premature: ☐☐

6. Abortuses: ☐☐

7. Living: ☐☐

8. Was a 50 g oral glucose challenge test done?
   - ☐ No
   - ☐ Yes
     - ↳ if yes, what were the test results?

   60 minutes: ☐☐ . ☐ mmol/L ☐ not done

9. Was a 75 g oral glucose tolerance test done?
   - ☐ No
   - ☐ Yes
     - ↳ if yes, what were the test results?

   Fasting: ☐☐ . ☐ mmol/L ☐ not done

   60 minutes: ☐☐ . ☐ mmol/L ☐ not done

   120 minutes: ☐☐ . ☐ mmol/L ☐ not done

10. Was GBS screening performed?
    - ☐ Unknown
    - ☐ No
      - ↳ if no, was there a reason to treat with GBS prophylaxis?
        - ☐ No
        - ☐ Yes – GBS bacteriuria
        - ☐ Yes – previous baby with GBS septicemia
    - ☐ Yes
      - ↳ if yes, what was the test result?
        - ☐ Positive
        - ☐ Negative
        - ☐ Unknown

11. Actual place of birth
    - ☐ Hospital
    - ☐ Home
    - ☐ Other: ____

12. Were any of the following antenatal risk factors present? (check all that apply)
    - ☐ None
    - ☐ Large for gestational age
    - ☐ Small for gestational age
    - ☐ Intrauterine growth restriction
    - ☐ PROM
    - ☐ PPROM
    - ☐ Pre-eclampsia
    - ☐ Gestational hypertension
    - ☐ Other: ____

13. Were any maternal antibiotics administered during the intrapartum period prior to the baby being born?
    - ☐ No
    - ☐ Yes
      - ↳ if yes, please complete the table on the following page.

Baby & Mi

PID: ☐☐ – ☐☐☐

Mother's Date of Birth: ☐☐☐☐ ☐☐ ☐☐
year month day

BIRTH

If antibiotics were administered, please indicate the following for each drug:

DRUG 1

Indication:
- ☐ Caesarean section
- ☐ GBS prophylaxis
- ☐ Signs and symptoms of maternal infection
- ☐ Other: ____________________

____
____
____

Drug:
- ☐ Penicillin G
- ☐ Cefazolin
- ☐ Clindamycin
- ☐ Erythromycin
- ☐ Ampicillin
- ☐ Vancomycin
- ☐ Other: ____________________

	Dosage:	Date and time:		Route IV	Route Oral
**Dose 1**	____________	☐☐☐☐ / ☐☐ / ☐☐ (year / month / day)	☐☐ : ☐☐ (24 hour clock)	☐	☐
**Dose 2**	____________	☐☐☐☐ / ☐☐ / ☐☐	☐☐ : ☐☐	☐	☐
**Dose 3**	____________	☐☐☐☐ / ☐☐ / ☐☐	☐☐ : ☐☐	☐	☐
**Dose 4**	____________	☐☐☐☐ / ☐☐ / ☐☐	☐☐ : ☐☐	☐	☐
**Dose 5**	____________	☐☐☐☐ / ☐☐ / ☐☐	☐☐ : ☐☐	☐	☐

DRUG 2 ☐ N/A

Indication:
- ☐ Caesarean section
- ☐ GBS prophylaxis
- ☐ Signs and symptoms of maternal infection
- ☐ Other: ____________________

____
____
____

Drug:
- ☐ Penicillin G
- ☐ Cefazolin
- ☐ Clindamycin
- ☐ Erythromycin
- ☐ Ampicillin
- ☐ Vancomycin
- ☐ Other: ____________________

	Dosage:	Date and time:		Route IV	Route Oral
**Dose 1**	____________	☐☐☐☐ / ☐☐ / ☐☐ (year / month / day)	☐☐ : ☐☐ (24 hour clock)	☐	☐
**Dose 2**	____________	☐☐☐☐ / ☐☐ / ☐☐	☐☐ : ☐☐	☐	☐
**Dose 3**	____________	☐☐☐☐ / ☐☐ / ☐☐	☐☐ : ☐☐	☐	☐
**Dose 4**	____________	☐☐☐☐ / ☐☐ / ☐☐	☐☐ : ☐☐	☐	☐
**Dose 5**	____________	☐☐☐☐ / ☐☐ / ☐☐	☐☐ : ☐☐	☐	☐

PID: ☐☐ – ☐☐☐

Mother's Date of Birth: ☐☐☐☐ ☐☐ ☐☐
year month day

BIRTH

DRUG 3 ❑ N/A

Indication:
- ❑ Caesarean section
- ❑ GBS prophylaxis
- ❑ Signs and symptoms of maternal infection
- ❑ Other: ____________

Drug:
- ❑ Penicillin G
- ❑ Cefazolin
- ❑ Clindamycin
- ❑ Erythromycin
- ❑ Ampicillin
- ❑ Vancomycin
- ❑ Other: ____________

	Dosage:	Date and time: (year / month / day)	24 hour clock	Route: IV	Oral
**Dose 1**	________	☐☐☐☐ / ☐☐ / ☐☐	☐☐ : ☐☐	❑	❑
**Dose 2**	________	☐☐☐☐ / ☐☐ / ☐☐	☐☐ : ☐☐	❑	❑
**Dose 3**	________	☐☐☐☐ / ☐☐ / ☐☐	☐☐ : ☐☐	❑	❑
**Dose 4**	________	☐☐☐☐ / ☐☐ / ☐☐	☐☐ : ☐☐	❑	❑
**Dose 5**	________	☐☐☐☐ / ☐☐ / ☐☐	☐☐ : ☐☐	❑	❑

DRUG 4 ❑ N/A

Indication:
- ❑ Caesarean section
- ❑ GBS prophylaxis
- ❑ Signs and symptoms of maternal infection
- ❑ Other: ____________

Drug:
- ❑ Penicillin G
- ❑ Cefazolin
- ❑ Clindamycin
- ❑ Erythromycin
- ❑ Ampicillin
- ❑ Vancomycin
- ❑ Other: ____________

	Dosage:	Date and time: (year / month / day)	24 hour clock	Route: IV	Oral
**Dose 1**	________	☐☐☐☐ / ☐☐ / ☐☐	☐☐ : ☐☐	❑	❑
**Dose 2**	________	☐☐☐☐ / ☐☐ / ☐☐	☐☐ : ☐☐	❑	❑
**Dose 3**	________	☐☐☐☐ / ☐☐ / ☐☐	☐☐ : ☐☐	❑	❑
**Dose 4**	________	☐☐☐☐ / ☐☐ / ☐☐	☐☐ : ☐☐	❑	❑
**Dose 5**	________	☐☐☐☐ / ☐☐ / ☐☐	☐☐ : ☐☐	❑	❑

14. Did the **mother** receive any antibiotics ***after*** the baby was born and ***prior*** to hospital discharge?
    - ☐ No
    - ☐ Yes
      ↳ if yes, please complete the following table:

DRUG 1

Primary indication: ____________________

Drug: ____________________

Dosage: ____________________

Route: ☐ IV ☐ Oral ☐ Other: ________

Start date and time: ☐☐☐☐ / ☐☐ / ☐☐ ☐☐ : ☐☐
year month day 24 hour clock

Stop date and time: ☐☐☐☐ / ☐☐ / ☐☐ ☐☐ : ☐☐
year month day 24 hour clock

DRUG 2 ☐ N/A

Primary indication: ____________________

Drug: ____________________

Dosage: ____________________

Route: ☐ IV ☐ Oral ☐ Other: ________

Start date and time: ☐☐☐☐ / ☐☐ / ☐☐ ☐☐ : ☐☐
year month day 24 hour clock

Stop date and time: ☐☐☐☐ / ☐☐ / ☐☐ ☐☐ : ☐☐
year month day 24 hour clock

BIRTH

15. Date and time of rupture of membranes: [ ][ ][ ][ ] / [ ][ ] / [ ][ ] [ ][ ] : [ ][ ] ❑ unknown
year month day 24 hour clock

16. Date and time of birth: [ ][ ][ ][ ] / [ ][ ] / [ ][ ] [ ][ ] : [ ][ ]
year month day 24 hour clock

17. Presentation at delivery:
    - ❑ Cephalic
    - ❑ Breech
    - ❑ Transverse/oblique
    - ❑ Unknown

18. Mode of delivery:
    - ❑ Vaginal
    - ❑ Caesarean section without labour
    - ❑ Caesarean section with labour
        - → if Caesarean section with labour, what was the dilation at time of CS? [ ][ ] cm

19. Status of baby at birth:
    - ❑ Alive
    - ❑ Stillborn

20. Gender:
    - ❑ Male
    - ❑ Female
    - ❑ Ambiguous

21. Apgar score: at 1 minute: [ ][ ] at 5 minutes: [ ][ ]

22. Birthweight: [ ][ ][ ][ ] grams

23. Length: [ ][ ] . [ ] cm

24. Head circumference: [ ][ ] . [ ] cm

25. Was the baby admitted to the NICU or ICU?
    - ❑ No
    - ❑ Yes
      - → if yes, what was the date and time of admission?

        [ ][ ][ ][ ] / [ ][ ] / [ ][ ]   [ ][ ] : [ ][ ]
        year month day 24 hour clock

      - → if yes, what was the date and time of discharge?

        [ ][ ][ ][ ] / [ ][ ] / [ ][ ]   [ ][ ] : [ ][ ]
        year month day 24 hour clock

26. Was the baby tested for hypoglycemia?
    - ❑ No
    - ❑ Yes
      - → if yes, were any glucose values less than 3.0 mmol/L?
        - ❑ No
        - ❑ Yes

27. Did the **baby** receive any antibiotics prior to discharge **other than** erythromycin prophylactic eye ointment?
    - ☐ No
    - ☐ Yes,
      ↳ if yes, please complete the following table:

DRUG 1

Primary indication: ____________________

Drug: ____________________

Dosage: ____________________

Route: ☐ IV ☐ Other: ____________________

Start date and time: ☐☐☐☐ / ☐☐ / ☐☐ ☐☐ : ☐☐
year month day 24 hour clock

Stop date and time: ☐☐☐☐ / ☐☐ / ☐☐ ☐☐ : ☐☐
year month day 24 hour clock

DRUG 2 ☐ N/A

Primary indication: ____________________

Drug: ____________________

Dosage: ____________________

Route: ☐ IV ☐ Other: ____________________

Start date and time: ☐☐☐☐ / ☐☐ / ☐☐ ☐☐ : ☐☐
year month day 24 hour clock

Stop date and time: ☐☐☐☐ / ☐☐ / ☐☐ ☐☐ : ☐☐
year month day 24 hour clock

28. What was the baby fed at first feeding?
    - ❑ Breast milk
    - ❑ Formula
    - ❑ Other:________________________________
    - ❑ Unknown

29. Was the baby fed any formula during hospital stay?
    - ❑ No hospital stay
    - ❑ No formula fed during hospital stay
    - ❑ Yes formula fed during hospital stay
    - ❑ Unknown

30. Date/ time of discharge of mother from hospital or midwife leaving home:

    [ ][ ][ ][ ] / [ ][ ] / [ ][ ]   [ ][ ] : [ ][ ]
    year month day 24 hour clock

31. Date/ time of discharge of baby from hospital or midwife leaving home:

    [ ][ ][ ][ ] / [ ][ ] / [ ][ ]   [ ][ ] : [ ][ ]
    year month day 24 hour clock

PID: ☐☐–☐☐☐

Mother's Date of Birth: ☐☐☐☐ ☐☐ ☐☐
year month day

Additional birth information

Instructions:

- **This form is to be completed by research personnel by telephone or in person at 12 weeks or the earliest contact postpartum (after June 15, 2015).**

1. Date: ☐☐☐☐ / ☐☐ / ☐☐
   year month day

2. When you were in labour, did you spend any time immersed in water?
   - ☐ No
   - ☐ Yes
     - → if yes, was your baby born in water?
       - ☐ No
       - ☐ Yes

3. During your baby's first 3 months of life, were there other children living in your home (part-time or full-time)?
   - ☐ No
   - ☐ Yes
     - → if yes, how many?
       ☐☐ children
     - → if yes, how many of the children attended school or daycare outside of your home during your baby's first 3 months?
       ☐☐ children

PID: [ ][ ] – [ ][ ][ ]

Mother's Date of Birth: [ ][ ][ ][ ] [ ][ ] [ ][ ]
year month day

Day 3

Instructions:

- **This form is to be completed when your baby is 3 days old.**
- **Mark only one box with an "X" for each question, unless instructed to do otherwise.**
- **Please use your study calendar to help you remember the information asked for.**
- **All of your answers will remain confidential.**
- **If you do not understand a question, please ask a research staff member by calling 905-525-9140 ext 22146 or by emailing babyandmistudy@gmail.com.**
- **Please return this form when you visit McMaster University Medical Center for your first study visit.**

1. What is today's date? [ ][ ][ ][ ] / [ ][ ] / [ ][ ]
year month day

2. How much did your baby weigh at your most recent visit with your midwife or doctor?

[ ][ ][ ][ ] grams or [ ][ ] lbs, [ ][ ] oz or ❑ I don't know

3. Have you *ever* breast fed your baby?
   - ❑ No
   - ❑ Yes
     - ↳ if yes, ***do you still*** breastfeed your baby?
       - ❑ No
         - ↳ if no, how old was your baby when you stopped breast feeding?

           [ ][ ] days
       - ❑ Yes
         - ↳ if yes, has your baby ***ever*** had anything other than breast milk or water?
           - ❑ No
           - ❑ Yes
             - ↳ if yes, how old was your baby when she or he first had anything other than breast milk or water?

               [ ][ ] days

4. Has your baby ***ever*** had any of the following?

	**No**	**Yes**
Formula	❑	❑
Soy milk	❑	❑
Goat's milk	❑	❑
Hypo-allergenic formula	❑	❑
Cow's milk	❑	❑
Another type of milk or formula:	❑	❑

____

5. ***Since you and your baby have been home***, has your baby needed to stay in the hospital?
   - ❑ No
   - ❑ Yes

Baby & Mi

PID: [ ][ ] – [ ][ ][ ]

Mother's Date of Birth: [ ][ ][ ][ ] [ ][ ] [ ][ ]
year month day

Day 3

---

6. ***Since you and your baby have been home***, has ***your baby*** had an infection that required antibiotic or antifungal (anti-yeast) treatment?
   - ❑ No
   - ❑ Yes→ if yes, please complete the following table:

DRUG 1

What was the type of infection? ❑ Thrush ❑ Ear infection ❑ Chest infection ❑ Urinary tract infection ❑ Other: ______________________________

What was the name of the drug? ______________________________

How was it given? ❑ Topical ❑ Oral ❑ Other: ______________________

What was the start date? [ ][ ][ ][ ] / [ ][ ] / [ ][ ]
year month day

What was the end date? [ ][ ][ ][ ] / [ ][ ] / [ ][ ] or ❑ Still currently in use
year month day

DRUG 2 ❑ Only one drug was given. (Do not complete the rest of the table)

What was the type of infection? ❑ Thrush ❑ Ear infection ❑ Chest infection ❑ Urinary tract infection ❑ Other: ______________________________

What was the name of the second drug? ______________________________

How was it given? ❑ Topical ❑ Oral ❑ Other: ______________________

What was the start date? [ ][ ][ ][ ] / [ ][ ] / [ ][ ]
year month day

What was the end date? [ ][ ][ ][ ] / [ ][ ] / [ ][ ] or ❑ Still currently in use
year month day

Day 3

7. Please list any other medications, vitamins, homeopathics or supplements that ***your baby*** has taken ***since birth***.

❑ None

Medication, vitamin or supplement name	Currently in Use? No	Currently in Use? Yes
a. ____	❑	❑
b. ____	❑	❑
c. ____	❑	❑
d. ____	❑	❑
e. ____	❑	❑

Baby & Mi

PID: ☐☐ – ☐☐☐

Mother's Date of Birth: ☐☐☐☐ ☐☐ ☐☐
year month day

Day 3

---

8. ***Since you and your baby have been home***, have ***you*** taken any antibiotic or antifungal (anti-yeast) medications?
   - ☐ No
   - ☐ Yes→ if yes, please complete the following table:

DRUG 1

What was the name of the drug? ______________________________

How was it given? ☐ Topical ☐ Oral ☐ Other: ____________

What was the start date? ☐☐☐☐ / ☐☐ / ☐☐
year month day

What was the end date? ☐☐☐☐ / ☐☐ / ☐☐ or ☐ Still currently in use
year month day

DRUG 2 ☐ Only one drug was taken. (Do not complete the rest of the table)

What was the name of the second drug? ______________________________

How was it given? ☐ Topical ☐ Oral ☐ Other: ____________

What was the start date? ☐☐☐☐ / ☐☐ / ☐☐
year month day

What was the end date? ☐☐☐☐ / ☐☐ / ☐☐ or ☐ Still currently in use
year month day

---

9. ***If you are currently breastfeeding,*** please list any other prescription medications that ***you*** have taken ***since your baby was born***.

☐ Not currently breastfeeding
☐ None

Medication name	Currently in Use? No	Yes
a. ______________________________	☐	☐
b. ______________________________	☐	☐
c. ______________________________	☐	☐
d. ______________________________	☐	☐
e. ______________________________	☐	☐

Baby & Mi

PID: ☐☐ – ☐☐☐

Mother's Date of Birth: ☐☐☐☐ ☐☐ ☐☐
year month day

DAY 10

Instructions:
- **This form is to be completed when your baby is 10 days old.**
- **Mark only one box with an "X" for each question, unless instructed to do otherwise.**
- **Please use your study calendar to help you remember the information asked for.**
- **All of your answers will remain confidential.**
- **If you do not understand a question, please ask a research staff member by calling 905-525-9140 ext 22146 or by emailing babyandmistudy@gmail.com.**
- **Please return this form when you visit McMaster University Medical Center for your first study visit.**

1. What is today's date? ☐☐☐☐ / ☐☐ / ☐☐
   year month day

2. How much did your baby weigh at your most recent visit with your midwife or doctor?

   ☐☐☐☐ grams or ☐☐ lbs, ☐☐ oz or ❑ I don't know

3. Have you *ever* breast fed your baby?
   - ❑ No
   - ❑ Yes
     - ↳ if yes, ***do you still*** breastfeed your baby?
       - ❑ No
         - ↳ if no, how old was your baby when you stopped breast feeding?

           ☐☐ days
       - ❑ Yes
         - ↳ if yes, has your baby ***ever*** had anything other than breast milk or water?
           - ❑ No
           - ❑ Yes
             - ↳ if yes, how old was your baby when she or he first had anything other than breast milk or water?

               ☐☐ days

DAY 10

---

4. Has your baby ***ever*** had any of the following?

	**No**	**Yes**
Formula	❑	❑
Soy milk	❑	❑
Goat's milk	❑	❑
Hypo-allergenic formula	❑	❑
Cow's milk	❑	❑
Another type of milk or formula:	❑	❑

____

5. ***Since you and your baby have been home***, has your baby needed to stay in the hospital?
   - ❑ No
   - ❑ Yes

6. How much time does your baby spend in sleep during the night (between 7 in the evening and 7 in the morning)? ☐☐ hours, ☐☐ minutes

7. How many times does your baby wake up during the night?

   ☐☐ times

8. How much time during the night does your baby spend in wakefulness (from 10 in the evening to 6 in the morning)? ☐☐ hours, ☐☐ minutes

9. Do you consider your baby's sleep to be a problem?
   - ❑ Not a problem at all
   - ❑ A small problem
   - ❑ A very serious problem

Baby & Mi

PID: ☐☐ – ☐☐☐

Mother's Date of Birth: ☐☐☐☐ ☐☐ ☐☐ (year, month, day)

DAY 10

10. ***Since you and your baby have been home***, has ***your baby*** had an infection that required antibiotic or antifungal (anti-yeast) treatment ***that you haven't told us about yet***?
   - ☐ No
   - ☐ Yes→ if yes, please complete the following table:

DRUG 1

What was the type of infection? ☐ Thrush ☐ Ear infection ☐ Chest infection ☐ Urinary tract infection ☐ Other: ___________________________

What was the name of the drug? ____

How was it given? ☐ Topical ☐ Oral ☐ Other: _____________________

What was the start date? ☐☐☐☐ / ☐☐ / ☐☐ (year, month, day)

What was the end date? ☐☐☐☐ / ☐☐ / ☐☐ (year, month, day) or ☐ Still currently in use

DRUG 2 ☐ Only one drug was given. (Do not complete the rest of the table)

What was the type of infection? ☐ Thrush ☐ Ear infection ☐ Chest infection ☐ Urinary tract infection ☐ Other: ___________________________

What was the name of the second drug? ____

How was it given? ☐ Topical ☐ Oral ☐ Other: _____________________

What was the start date? ☐☐☐☐ / ☐☐ / ☐☐ (year, month, day)

What was the end date? ☐☐☐☐ / ☐☐ / ☐☐ (year, month, day) or ☐ Still currently in use

11. Please list any other medications, vitamins, homeopathics or supplements that ***your baby*** has taken ***since birth***.

☐ None

Medication, vitamin or supplement name	Currently in Use? No	Currently in Use? Yes
a. ________________________________	☐	☐
b. ________________________________	☐	☐
c. ________________________________	☐	☐
d. ________________________________	☐	☐
e. ________________________________	☐	☐

PID: ☐☐–☐☐☐

Mother's Date of Birth: ☐☐☐☐ ☐☐ ☐☐
year month day

DAY 10

---

12. ***Since you and your baby have been home***, have ***you*** taken any antibiotic or antifungal (anti-yeast) medications ***that you haven't told us about yet***?
   - ☐ No
   - ☐ Yes→ if yes, please complete the following table:

DRUG 1

What was the name of the drug? ____________________

How was it given? ☐ Topical ☐ Oral ☐ Other: ____________________

What was the start date? ☐☐☐☐ / ☐☐ / ☐☐
year month day

What was the end date? ☐☐☐☐ / ☐☐ / ☐☐ or ☐ Still currently in use
year month day

DRUG 2 ☐ Only one drug was taken. (Do not complete the rest of the table)

What was the name of the second drug? ____________________

How was it given? ☐ Topical ☐ Oral ☐ Other: ____________________

What was the start date? ☐☐☐☐ / ☐☐ / ☐☐
year month day

What was the end date? ☐☐☐☐ / ☐☐ / ☐☐ or ☐ Still currently in use
year month day

Baby & Mi

PID: [ ][ ] – [ ][ ][ ]

Mother's Date of Birth: [ ][ ][ ][ ] [ ][ ] [ ][ ]
year month day

DAY 10

---

13. **If you are currently breastfeeding**, please list any other prescription medications that ***you*** have taken ***since your baby was born***.

    ❑ Not currently breastfeeding
    ❑ None

Medication name	Currently in Use? No	Currently in Use? Yes
a. ____	❑	❑
b. ____	❑	❑
c. ____	❑	❑
d. ____	❑	❑
e. ____	❑	❑

Baby & Mi

PID: ☐☐ – ☐☐☐

Mother's Date of Birth: ☐☐☐☐ ☐☐ ☐☐
year month day

6 WEEKS

Instructions:
- **This form is to be completed when your baby is 6 weeks old.**
- **Mark only one box with an "X" for each question, unless instructed to do otherwise.**
- **Please use your study calendar to help you remember the information asked for.**
- **All of your answers will remain confidential.**
- **If you do not understand a question, please ask a research staff member by calling 905-525-9140 ext 22146 or by emailing babyandmistudy@gmail.com.**
- **Please return this form when you visit McMaster University Medical Center for your first study visit.**

1. What is today's date? ☐☐☐☐ / ☐☐ / ☐☐
year month day

2. How much did your baby weigh at your most recent visit with your midwife or doctor?

☐☐☐☐ grams or ☐☐ lbs, ☐☐ oz or ❑ I don't know

3. Have you *ever* breast fed your baby?
   - ❑ No
   - ❑ Yes
     ↳ if yes, ***do you still*** breastfeed your baby?
     - ❑ No
       ↳ if no, how old was your baby when you stopped breast feeding?
       ☐☐ weeks
       (fill in "0" if your baby was less than 1 week old)
     - ❑ Yes
       ↳ if yes, has your baby ***ever*** had anything other than breast milk or water?
       - ❑ No
       - ❑ Yes
         ↳ if yes, how old was your baby when he or she first had anything other than breast milk or water?
         ☐☐ weeks
         (fill in "0" if your baby was less than 1 week old)

6 WEEKS

---

4. Has your baby ***ever*** had any of the following?

	No	Yes	Current Use
Formula	❑	❑ →	☐☐ times per week
Soy milk	❑	❑ →	☐☐ times per week
Goat's milk	❑	❑ →	☐☐ times per week
Hypo-allergenic formula	❑	❑ →	☐☐ times per week
Cow's milk	❑	❑ →	☐☐ times per week
Another type of milk or formula: ________________________________	❑	❑ →	☐☐ times per week

Baby & Mi

PID: ☐☐ – ☐☐☐

Mother's Date of Birth: ☐☐☐☐ ☐☐ ☐☐
year month day

6 WEEKS

5. Has your baby ***ever*** had any of the following foods or beverages?

	No	Yes	Current Use
Rice cereal	❑	❑ →	☐☐ times per week
Other cereal	❑	❑ →	☐☐ times per week
Bread or toast	❑	❑ →	☐☐ times per week
Baby cookies	❑	❑ →	☐☐ times per week
Baked goods	❑	❑ →	☐☐ times per week
Dairy products	❑	❑ →	☐☐ times per week
Egg yolk	❑	❑ →	☐☐ times per week
Egg white	❑	❑ →	☐☐ times per week
Meat	❑	❑ →	☐☐ times per week
Fish	❑	❑ →	☐☐ times per week
Raw vegetables	❑	❑ →	☐☐ times per week
Potato	❑	❑ →	☐☐ times per week
Cooked vegetables	❑	❑ →	☐☐ times per week
Legumes	❑	❑ →	☐☐ times per week
Soy products (i.e. tofu)	❑	❑ →	☐☐ times per week
Raw fruit	❑	❑ →	☐☐ times per week
Cooked fruit	❑	❑ →	☐☐ times per week

PID: ☐☐ – ☐☐☐

Mother's Date of Birth: ☐☐☐☐ (year) ☐☐ (month) ☐☐ (day)

6 WEEKS

	No	Yes	Current Use
Pop	❑	❑ →	☐☐ times per week
Other fizzy drinks	❑	❑ →	☐☐ times per week
Apple juice	❑	❑ →	☐☐ times per week
Other fruit drinks	❑	❑ →	☐☐ times per week
Herbal drink	❑	❑ →	☐☐ times per week
Unpasteurized milk	❑	❑ →	☐☐ times per week
Gripe water	❑	❑ →	☐☐ times per week
Tea (herbal)	❑	❑ →	☐☐ times per week
Tea (caffeinated)	❑	❑ →	☐☐ times per week
Coffee	❑	❑ →	☐☐ times per week
A little alcohol	❑	❑ →	☐☐ times per week
Potato chips	❑	❑ →	☐☐ times per week
Other salty snacks	❑	❑ →	☐☐ times per week
Chocolate	❑	❑ →	☐☐ times per week
Sweets	❑	❑ →	☐☐ times per week
Peanuts/ peanut butter	❑	❑ →	☐☐ times per week
Tree nuts/ tree nut butter (i.e. almond, cashew)	❑	❑ →	☐☐ times per week

6 WEEKS

6. Please indicate if your baby ***currently*** has any of the following feeding behaviours:

	No	Yes	
Slow feeding	❑	❑	
Taking only small quantities at each feed	❑	❑	
Choking	❑	❑	
Hungry/ not satisfied	❑	❑	
Refused to take breast milk	❑	❑	❑ **Not applicable**
Refused to take other milk	❑	❑	❑ **Not applicable**
Refused to take solids	❑	❑	❑ **Not applicable**
Can not establish feeding routine	❑	❑	

7. Are you ***currently*** having difficulties feeding your baby?
   - ❑ No, no difficulties
   - ❑ Yes, some difficulties
   - ❑ Yes, great difficulties

8. Does your baby typically have ***at least one*** bowel movement every day?
   - ❑ No
     ↳ if no, how many bowel movements does your baby have in a ***typical week***?
     ☐☐ bowel movements
   - ❑ Yes
     ↳ if yes, how many bowel movements does your baby have in a ***typical day***?
     ☐☐ bowel movements

Baby & Mi

PID: ☐☐–☐☐☐

Mother's Date of Birth: ☐☐☐☐ (year) ☐☐ (month) ☐☐ (day)

6 WEEKS

---

9. ***Since your baby was 10 days old***, has ***she or he*** had an infection that required antibiotic or antifungal (anti-yeast) treatment?
   - ❑ No
   - ❑ Yes→ if yes, please complete the following table:

DRUG 1

What was the type of infection? ❑ Thrush ❑ Ear infection ❑ Chest infection ❑ Urinary tract infection ❑ Other: ____________________________

What was the name of the drug? ____

How was it given? ❑ Topical ❑ Oral ❑ Other: _____________________

What was the start date? ☐☐☐☐ / ☐☐ / ☐☐ (year / month / day)

What was the end date? ☐☐☐☐ / ☐☐ / ☐☐ (year / month / day) or ❑ Still currently in use

---

DRUG 2 ❑ Only one drug was given. (Do not complete the rest of the table)

What was the type of infection? ❑ Thrush ❑ Ear infection ❑ Chest infection ❑ Urinary tract infection ❑ Other: ____________________________

What was the name of the second drug? ____

How was it given? ❑ Topical ❑ Oral ❑ Other: _____________________

What was the start date? ☐☐☐☐ / ☐☐ / ☐☐ (year / month / day)

What was the end date? ☐☐☐☐ / ☐☐ / ☐☐ (year / month / day) or ❑ Still currently in use

---

10. Please list any other medications, vitamins, homeopathics or supplements that ***your baby*** has taken ***since he or she was 10 days old***.

❑ None

Medication, vitamin or supplement name	Currently in Use? No	Currently in Use? Yes
a. ____	❑	❑
b. ____	❑	❑
c. ____	❑	❑
d. ____	❑	❑
e. ____	❑	❑

11. ***Since your baby was 10 days old***, has she or he needed to stay in the hospital?
❑ No
❑ Yes

PID: ☐☐ – ☐☐☐

Mother's Date of Birth: ☐☐☐☐ (year) ☐☐ (month) ☐☐ (day)

6 WEEKS

---

12. ***Since your baby was 10 days old***, have ***you*** taken any antibiotic or antifungal (anti-yeast) medications?
    - ❑ No
    - ❑ Yes→ if yes, please complete the following table:

DRUG 1

What was the name of the drug? ______________________________

How was it given? ❑ Topical ❑ Oral ❑ Other: ____________

What was the start date? ☐☐☐☐ / ☐☐ / ☐☐ (year / month / day)

What was the end date? ☐☐☐☐ / ☐☐ / ☐☐ (year / month / day) or ❑ Still currently in use

---

DRUG 2 ❑ Only one drug was taken. (Do not complete the rest of the table)

What was the name of the second drug? ______________________________

How was it given? ❑ Topical ❑ Oral ❑ Other: ____________

What was the start date? ☐☐☐☐ / ☐☐ / ☐☐ (year / month / day)

What was the end date? ☐☐☐☐ / ☐☐ / ☐☐ (year / month / day) or ❑ Still currently in use

---

13. ***If you are currently breastfeeding,*** Please list any other prescription medications that ***you*** have taken ***since your baby was 10 days old***.

    ☐ Not currently breastfeeding
    ☐ None

Medication name	Currently in Use? No	Yes
a. ______________________	☐	☐
b. ______________________	☐	☐
c. ______________________	☐	☐
d. ______________________	☐	☐
e. ______________________	☐	☐

14. How much time does your baby spend in sleep during the night (between 7 in the evening and 7 in the morning)? ☐☐ hours, ☐☐ minutes

15. How many times does your baby wake up during the night?

    ☐☐ times

16. How much time during the night does your baby spend in wakefulness (from 10 in the evening to 6 in the morning)? ☐☐ hours, ☐☐ minutes

17. Do you consider your baby's sleep to be a problem?
    ☐ Not a problem at all
    ☐ A small problem
    ☐ A very serious problem

18. Has your baby been diagnosed with asthma or reactive airway disease by your doctor?
    ☐ Yes
    ☐ No

6 WEEKS

---

19. Does your baby take any puffers or breathing medications?
    - ☐ Yes
    - ☐ No

20. Have you been told by your doctor that your baby has eczema?
    - ☐ Yes
    - ☐ No

21. Has your baby ***ever*** experienced any of the following?: (check all that apply)
    - ☐ Itchy rashes
    - ☐ Itchy rashes that don't go away on the face, knuckles, elbows or knees
    - ☐ Rash on the nose, mouth or diaper area
    - ☐ Dry, thickened or scaly skin or more skin creases in the palms than usual
    - ☐ Cracked skin around the ear
    - ☐ Scaly scalp that won't go away
    - ☐ Red dots surrounding hair follicles
    - ☐ None of the above

22. Have you ***ever*** had any concerns that your baby may have an allergy?
    - ☐ No
    - ☐ Yes, I/we suspect(ed) an allergy

      ↳ if yes, please describe what the allergic substance was thought to be and what made you suspect and allergy:

      ____

      ____

      ____

    - ☐ Yes, an allergy was confirmed by the doctor

      ↳ if yes, please indicate what the allergic substance(s) is/are:

      ____

      ____

      ____

Baby & Mi

PID: ☐☐ – ☐☐☐

Mother's Date of Birth: ☐☐☐☐ ☐☐ ☐☐
year month day

12 WEEKS – PART 1

Instructions:
- **This form is to be completed by research personnel at the 12 week study visit.**
- **Record the measurements indicated below and attach this form to "Part 2" completed by the participant.**

1. Date and time of visit: ☐☐☐☐ / ☐☐ / ☐☐ ☐☐ : ☐☐
   year month day 24 hour clock

2. Head circumference: ☐☐ . ☐ cm

3. Tricep skinfold thickness:

   Measurement 1: ☐☐ . ☐ cm

   Measurement 2: ☐☐ . ☐ cm

   Measurement 3: ☐☐ . ☐ cm

4. Subscapular skinfold thickness:

   Measurement 1: ☐☐ . ☐ cm

   Measurement 2: ☐☐ . ☐ cm

   Measurement 3: ☐☐ . ☐ cm

5. Bicep skinfold thickness:

   Measurement 1: ☐☐ . ☐ cm

   Measurement 2: ☐☐ . ☐ cm

   Measurement 3: ☐☐ . ☐ cm

12 WEEKS – PART 1

6. Mid-arm circumference:

   Measurement 1: __ __ . __ cm

   Measurement 2: __ __ . __ cm

   Measurement 3: __ __ . __ cm

7. Suprailiac skinfold thickness:

   Measurement 1: __ __ . __ cm

   Measurement 2: __ __ . __ cm

   Measurement 3: __ __ . __ cm

8. Hip circumference:

   Measurement 1: __ __ . __ cm

   Measurement 2: __ __ . __ cm

   Measurement 3: __ __ . __ cm

9. Abdominal circumference:

   Measurement 1: __ __ . __ cm

   Measurement 2: __ __ . __ cm

   Measurement 3: __ __ . __ cm

10. Length: __ __ __ . __ cm

Baby & Mi

PID: __ __ – __ __ __

Mother's Date of Birth: __ __ __ __ / __ __ / __ __
year month day

12 WEEKS – PART 1

---

11. Percent fat: __ __ . __ %
12. Percent fat free mass: __ __ . __ %
13. Fat mass: __ __ . __ __ __ __ kg
14. Fat free mass: __ __ . __ __ __ __ kg
15. Body mass: __ __ . __ __ __ __ kg
16. Body volume: __ __ . __ __ __ __ L
17. Body density: __ __ . __ __ __ __ kg/L
18. Fat mass density: __ __ . __ __ __ __ kg/L
19. Fat free mass density: __ __ . __ __ __ __ kg/L
20. Body surface area: __ __ __ __ . __ cm^2
21. Thoracic gas volume: __ __ . __ __ __ __ L
22. The PEA POD test was:
    - ❑ Completed
    - ❑ Begun but terminated early
    - ❑ Not attempted because baby exceeded capacity
    - ❑ Not attempted because parent declined
23. Were stool samples received?
    - ❑ No
    - ❑ Yes
      - ↳ if yes, indicate the following:

	Date Collected
**Day 3**	__ __ __ __ / __ __ / __ __ (year / month / day)
**Day 10**	__ __ __ __ / __ __ / __ __
**6 Weeks**	__ __ __ __ / __ __ / __ __
**12 Weeks**	__ __ __ __ / __ __ / __ __

Baby&Mi

PID: ☐☐ – ☐☐☐

Mother's Date of Birth: ☐☐☐☐ ☐☐ ☐☐
year month day

12 WEEKS – PART 2

Instructions:
- **Mark only one box with an "X" for each question, unless instructed to do otherwise.**
- **Please use your study calendar to help you remember the information asked for.**
- **All of your answers will remain confidential.**
- **If you do not understand a question, please ask a research staff member.**

1. Have you ***ever*** breast fed your baby?
   - ☐ No
   - ☐ Yes
     - ↳ if yes, ***do you still*** breastfeed your baby?
       - ☐ No
         - ↳ if no, how old was your baby when you stopped breast feeding?

           ☐☐ weeks

           (fill in "0" if your baby was less than 1 week old)
       - ☐ Yes
         - ↳ if yes, has your baby ***ever*** had anything other than breast milk or water?
           - ☐ No
           - ☐ Yes
             - ↳ if yes, how old was your baby when he or she first had anything other than breast milk or water?

               ☐☐ weeks

               (fill in "0" if your baby was less than 1 week old)

2. Has your baby ***ever*** had any of the following?

	**No**	**Yes**	**Current Use**
Formula	☐	☐ →	☐☐ times per week
Soy milk	☐	☐ →	☐☐ times per week
Goat's milk	☐	☐ →	☐☐ times per week
Hypo-allergenic formula	☐	☐ →	☐☐ times per week
Cow's milk	☐	☐ →	☐☐ times per week
Another type of milk or formula: ______________________	☐	☐ →	☐☐ times per week

Baby & Mi

PID: ☐☐ – ☐☐☐

Mother's Date of Birth: ☐☐☐☐ ☐☐ ☐☐
year month day

12 WEEKS – PART 2

---

3. Has your baby ***ever*** had any of the following foods or beverages?

	No	Yes	Current Use
Rice cereal	☐	☐ →	☐☐ times per week
Other cereal	☐	☐ →	☐☐ times per week
Bread or toast	☐	☐ →	☐☐ times per week
Baby cookies	☐	☐ →	☐☐ times per week
Baked goods	☐	☐ →	☐☐ times per week
Dairy products	☐	☐ →	☐☐ times per week
Egg yolk	☐	☐ →	☐☐ times per week
Egg white	☐	☐ →	☐☐ times per week
Meat	☐	☐ →	☐☐ times per week
Fish	☐	☐ →	☐☐ times per week
Raw vegetables	☐	☐ →	☐☐ times per week
Potato	☐	☐ →	☐☐ times per week
Cooked vegetables	☐	☐ →	☐☐ times per week
Legumes	☐	☐ →	☐☐ times per week
Soy products (i.e. tofu)	☐	☐ →	☐☐ times per week
Raw fruit	☐	☐ →	☐☐ times per week
Cooked fruit	☐	☐ →	☐☐ times per week

PID: ☐☐ – ☐☐☐

Mother's Date of Birth: ☐☐☐☐ ☐☐ ☐☐
year month day

12 WEEKS – PART 2

	No	Yes	Current Use
Pop	❑	❑ →	☐☐ times per week
Other fizzy drinks	❑	❑ →	☐☐ times per week
Apple juice	❑	❑ →	☐☐ times per week
Other fruit drinks	❑	❑ →	☐☐ times per week
Herbal drink	❑	❑ →	☐☐ times per week
Unpasteurized milk	❑	❑ →	☐☐ times per week
Gripe water	❑	❑ →	☐☐ times per week
Tea (herbal)	❑	❑ →	☐☐ times per week
Tea (caffeinated)	❑	❑ →	☐☐ times per week
Coffee	❑	❑ →	☐☐ times per week
A little alcohol	❑	❑ →	☐☐ times per week
Potato chips	❑	❑ →	☐☐ times per week
Other salty snacks	❑	❑ →	☐☐ times per week
Chocolate	❑	❑ →	☐☐ times per week
Sweets	❑	❑ →	☐☐ times per week
Peanuts/ peanut butter	❑	❑ →	☐☐ times per week
Tree nuts/ tree nut butter (i.e. almond, cashew)	❑	❑ →	☐☐ times per week

PID: ☐☐ – ☐☐☐

Mother's Date of Birth: ☐☐☐☐ ☐☐ ☐☐
year month day

12 WEEKS – PART 2

---

4. Please indicate if your baby ***currently*** has any of the following feeding behaviours:

	No	Yes	
Slow feeding	❑	❑	
Taking only small quantities at each feed	❑	❑	
Choking	❑	❑	
Hungry/ not satisfied	❑	❑	
Refused to take breast milk	❑	❑	❑ **Not applicable**
Refused to take other milk	❑	❑	❑ **Not applicable**
Refused to take solids	❑	❑	❑ **Not applicable**
Can not establish feeding routine	❑	❑	

5. Are you ***currently*** having difficulties feeding your baby?
   - ❑ No, no difficulties
   - ❑ Yes, some difficulties
   - ❑ Yes, great difficulties

6. ***During the past week***, how often did your baby usually spit-up (anything coming out of the mouth) during a 24-hour period?
   - ❑ Less than once
   - ❑ 1 to 3 times
   - ❑ 4 to 6 times
   - ❑ More than 6 times

7. ***During the past week***, how much did your baby usually spit-up (anything coming out of the mouth) during a typical episode?
   - ❑ Did not spit up
   - ❑ Less than 1 tablespoonful
   - ❑ 1 tablespoonful to 2 ounces
   - ❑ More than 2 ounces to half the feeding
   - ❑ More than half the feeding

8. ***During the past week***, how often did spitting up (anything coming out of the mouth) seem to be uncomfortable for your baby, for example, crying, fussing, irritability, etc?
   - ❑ Never
   - ❑ Rarely
   - ❑ Sometimes
   - ❑ Often
   - ❑ Always

9. ***During the past week***, how often did your baby refuse a feeding even when hungry?
   - ❑ Never
   - ❑ Rarely
   - ❑ Sometimes
   - ❑ Often
   - ❑ Always

10. ***During the past week***, how often did your baby stop eating soon after starting even when hungry?
    - ❑ Never
    - ❑ Rarely
    - ❑ Sometimes
    - ❑ Often
    - ❑ Always

11. ***During the past week***, did your baby cry a lot during or within 1 hour after feedings?
    - ❑ Never
    - ❑ Rarely
    - ❑ Sometimes
    - ❑ Often
    - ❑ Always

12. ***During the past week***, did your baby cry or fuss more than usual?
    - ❑ Never
    - ❑ Rarely
    - ❑ Sometimes
    - ❑ Often
    - ❑ Always

PID: ☐☐ – ☐☐☐

Mother's Date of Birth: ☐☐☐☐ ☐☐ ☐☐
year month day

12 WEEKS – PART 2

---

13. ***During the past week***, on average how long did your baby cry or fuss during a 24 hour period?
    - ❑ Less than 10 minutes
    - ❑ 10 minutes to 1 hour
    - ❑ More than 1 hour but less than 3 hours
    - ❑ 3 or more hours

14. ***During the past week***, how often did your baby have hiccups?
    - ❑ Never
    - ❑ Rarely
    - ❑ Sometimes
    - ❑ Often
    - ❑ Always

15. ***During the past week***, how often did your baby have episodes of arching back?
    - ❑ Never
    - ❑ Rarely
    - ❑ Sometimes
    - ❑ Often
    - ❑ Always

16. ***During the past week***, has your baby stopped breathing while awake or struggled to breathe?
    - ❑ No
    - ❑ Yes

17. ***During the past week***, has your baby turned blue or purple?
    - ❑ No
    - ❑ Yes

12 WEEKS – PART 2

18. ***In the last month***, have you noted your baby straining for 10 minutes or longer before successful passage of stool?
    - ☐ No
    - ☐ Yes
      ↳ if yes, how many times per week?
      ☐☐ times

19. Does your baby typically have ***at least one*** bowel movement every day?
    - ☐ No
      ↳ if no, how many bowel movements does your baby have in a ***typical week***?
      ☐☐ bowel movements
    - ☐ Yes
      ↳ if yes, how many bowel movements does your baby have in a ***typical day***?
      ☐☐ bowel movements

20. ***In the last month***, has your baby had any episodes of abdominal pain or discomfort?
    - ☐ No
    - ☐ Yes

21. ***Since your last study visit***, has your baby needed to stay in the hospital?
    - ☐ No
    - ☐ Yes

Baby & Mi

PID: ☐☐-☐☐☐

Mother's Date of Birth: ☐☐☐☐ ☐☐ ☐☐
year month day

12 WEEKS – PART 2

22. ***Since your last study visit***, has ***your baby*** had an infection that required antibiotic or antifungal (anti-yeast) treatment?
    - ❑ No
    - ❑ Yes→ if yes, please complete the following table:

DRUG 1

What was the type of infection? ❑ Thrush ❑ Ear infection ❑ Chest infection ❑ Urinary tract infection ❑ Other: ____________________________

What was the name of the drug? ____

How was it given? ❑ Topical ❑ Oral ❑ Other: _____________________

What was the start date? ☐☐☐☐ / ☐☐ / ☐☐
year month day

What was the end date? ☐☐☐☐ / ☐☐ / ☐☐ or ❑ Still currently in use
year month day

DRUG 2 ❑ Only one drug was given. (Do not complete the rest of the table)

What was the type of infection? ❑ Thrush ❑ Ear infection ❑ Chest infection ❑ Urinary tract infection ❑ Other: ____________________________

What was the name of the second drug? ____

How was it given? ❑ Topical ❑ Oral ❑ Other: _____________________

What was the start date? ☐☐☐☐ / ☐☐ / ☐☐
year month day

What was the end date? ☐☐☐☐ / ☐☐ / ☐☐ or ❑ Still currently in use
year month day

23. Please list any other medications, vitamins, homeopathics or supplements that ***your baby*** has taken ***since your last study visit***.

❑ None

Medication, vitamin or supplement name	Currently in Use? No	Currently in Use? Yes
a. ______________________________	❑	❑
b. ______________________________	❑	❑
c. ______________________________	❑	❑
d. ______________________________	❑	❑
e. ______________________________	❑	❑

12 WEEKS – PART 2

24. ***Since your last study visit***, have ***you*** taken any antibiotic or antifungal (anti-yeast) medications?
    - ☐ No
    - ☐ Yes→ if yes, please complete the following table:

DRUG 1

What was the name of the drug? ____________________

How was it given? ☐ Topical ☐ Oral ☐ Other: ____________________

What was the start date? ☐☐☐☐ / ☐☐ / ☐☐
year month day

What was the end date? ☐☐☐☐ / ☐☐ / ☐☐ or ☐ Still currently in use
year month day

DRUG 2 ☐ Only one drug was taken. (Do not complete the rest of the table)

What was the name of the second drug? ____________________

How was it given? ☐ Topical ☐ Oral ☐ Other: ____________________

What was the start date? ☐☐☐☐ / ☐☐ / ☐☐
year month day

What was the end date? ☐☐☐☐ / ☐☐ / ☐☐ or ☐ Still currently in use
year month day

12 WEEKS – PART 2

---

25. ***If you are currently breastfeeding,*** please list any other prescription medications that ***you*** have taken ***since your last study visit***.

❑ Not currently breastfeeding
❑ None

Medication name	Currently in Use? No	Currently in Use? Yes
a. ______________________	❑	❑
b. ______________________	❑	❑
c. ______________________	❑	❑
d. ______________________	❑	❑
e. ______________________	❑	❑

26. How much time does your baby spend in sleep during the night (between 7 in the evening and 7 in the morning)? ☐☐ hours, ☐☐ minutes

27. How many times does your baby wake up during the night?

☐☐ times

28. How much time during the night does your baby spend in wakefulness (from 10 in the evening to 6 in the morning)? ☐☐ hours, ☐☐ minutes

29. Do you consider your baby's sleep to be a problem?
❑ Not a problem at all
❑ A small problem
❑ A very serious problem

30. Has your baby been diagnosed with asthma or reactive airway disease by your doctor?
❑ Yes
❑ No

PID: ☐☐ – ☐☐☐

Mother's Date of Birth: ☐☐☐☐ ☐☐ ☐☐
year month day

12 WEEKS – PART 2

---

31. Does your baby take any puffers or breathing medications?
    - ❑ Yes
    - ❑ No

32. Have you been told by your doctor that your baby has eczema?
    - ❑ Yes
    - ❑ No

33. ***In the last month***, has your baby experienced any of the following?: (check all that apply)
    - ❑ Itchy rashes
    - ❑ Itchy rashes that don't go away on the face, knuckles, elbows or knees
    - ❑ Rash on the nose, mouth or diaper area
    - ❑ Dry, thickened or scaly skin or more skin creases in the palms than usual
    - ❑ Cracked skin around the ear
    - ❑ Scaly scalp that won't go away
    - ❑ Red dots surrounding hair follicles
    - ❑ None of the above

34. Have you ***ever*** had any concerns that your baby may have an allergy?
    - ❑ No
    - ❑ Yes, I/we suspect(ed) an allergy
      ↳ if yes, please describe what the allergic substance was thought to be and what made you suspect and allergy:

      ____

      ____

      ____

    - ❑ Yes, an allergy was confirmed by the doctor
      ↳ if yes, please indicate what the allergic substance(s) is/are:

      ____

      ____

      ____

Baby & Mi

PID: ☐☐ – ☐☐☐

Mother's Date of Birth: ☐☐☐☐ ☐☐ ☐☐
year month day

PEA POD QUESTIONNAIRE

Instructions:
- **Mark only one box with an "X" for each question, unless instructed to do otherwise.**
- **If you do not understand a question, please ask a research staff member.**

1. What were your feelings about the PEA POD prior to its use?
   - ☐ I was concerned/ worried
   - ☐ I was curious/ interested
   - ☐ I was indifferent
   - ☐ I was uncomfortable
   - ☐ Other: ____

2. Is your experience with the PEA POD what you expected based on the information that we provided to you?
   - ☐ Yes
   - ☐ No
   - ☐ I had no expectations about the PEA POD

3. How comfortable were you during the use of the PEA POD?
   - ☐ Very comfortable
   - ☐ Comfortable
   - ☐ Neither comfortable nor uncomfortable
   - ☐ Uncomfortable
   - ☐ Very uncomfortable

4. How comfortable do you think your baby was while inside the PEA POD?
   - ☐ Very comfortable
   - ☐ Comfortable
   - ☐ Neither comfortable nor uncomfortable
   - ☐ Uncomfortable
   - ☐ Very uncomfortable

5. Additional comments, questions or concerns about the PEA POD:

   ____

   ____

   ____

   ____

PID: ☐☐ – ☐☐☐

Mother's Date of Birth: ☐☐☐☐ ☐☐ ☐☐
year month day

4 MONTHS

Instructions:
- **This form is to be completed by research personnel through telephone contact with participants at 4 months postpartum.**
- **Please refer to previous forms to appropriately prompt and ensure consistency for questions 3-5, 9, 12 and 21.**

1. Was the 4 month follow up completed?
   - ☐ No
   - ☐ Yes

2. Date: ☐☐☐☐ / ☐☐ / ☐☐
   year month day

3. Have you ***ever*** breast fed your baby?
   - ☐ No
   - ☐ Yes
     - ↳ if yes, ***do you still*** breastfeed your baby?
       - ☐ No
         - ↳ if no, how old was your baby when you stopped breast feeding?

           ☐☐ weeks

           (fill in "0" if your baby was less than 1 week old)
       - ☐ Yes
         - ↳ if yes, has your baby ***ever*** had anything other than breast milk or water?
           - ☐ No
           - ☐ Yes
             - ↳ if yes, how old was your baby when he or she first had anything other than breast milk or water?

               ☐☐ weeks

               (fill in "0" if your baby was less than 1 week old)

Baby & Mi

PID: [ ][ ] – [ ][ ][ ]

Mother's Date of Birth: [ ][ ][ ][ ] year [ ][ ] month [ ][ ] day

4 MONTHS

---

4. Has your baby ***ever*** had any of the following?

	No	Yes	Current Use
Formula	❑	❑ →	[ ][ ] times per week
Soy milk	❑	❑ →	[ ][ ] times per week
Goat's milk	❑	❑ →	[ ][ ] times per week
Hypo-allergenic formula	❑	❑ →	[ ][ ] times per week
Cow's milk	❑	❑ →	[ ][ ] times per week
Another type of milk or formula: ________________________________	❑	❑ →	[ ][ ] times per week

Baby & Mi

PID: ☐☐ – ☐☐☐

Mother's Date of Birth: ☐☐☐☐ ☐☐ ☐☐
year month day

4 MONTHS

---

5. Has your baby ***ever*** had any of the following foods or beverages?

	No	Yes	Current Use
Rice cereal	❑	❑ →	☐☐ times per week
Other cereal	❑	❑ →	☐☐ times per week
Bread or toast	❑	❑ →	☐☐ times per week
Baby cookies	❑	❑ →	☐☐ times per week
Baked goods	❑	❑ →	☐☐ times per week
Dairy products	❑	❑ →	☐☐ times per week
Egg yolk	❑	❑ →	☐☐ times per week
Egg white	❑	❑ →	☐☐ times per week
Meat	❑	❑ →	☐☐ times per week
Fish	❑	❑ →	☐☐ times per week
Raw vegetables	❑	❑ →	☐☐ times per week
Potato	❑	❑ →	☐☐ times per week
Cooked vegetables	❑	❑ →	☐☐ times per week
Legumes	❑	❑ →	☐☐ times per week
Soy products (i.e. tofu)	❑	❑ →	☐☐ times per week
Raw fruit	❑	❑ →	☐☐ times per week
Cooked fruit	❑	❑ →	☐☐ times per week

Baby & Mi

PID: ☐☐ – ☐☐☐

Mother's Date of Birth: ☐☐☐☐ ☐☐ ☐☐
year month day

4 MONTHS

	No	Yes	Current Use
Pop	❑	❑ →	☐☐ times per week
Other fizzy drinks	❑	❑ →	☐☐ times per week
Apple juice	❑	❑ →	☐☐ times per week
Other fruit drinks	❑	❑ →	☐☐ times per week
Herbal drink	❑	❑ →	☐☐ times per week
Unpasteurized milk	❑	❑ →	☐☐ times per week
Gripe water	❑	❑ →	☐☐ times per week
Tea (herbal)	❑	❑ →	☐☐ times per week
Tea (caffeinated)	❑	❑ →	☐☐ times per week
Coffee	❑	❑ →	☐☐ times per week
A little alcohol	❑	❑ →	☐☐ times per week
Potato chips	❑	❑ →	☐☐ times per week
Other salty snacks	❑	❑ →	☐☐ times per week
Chocolate	❑	❑ →	☐☐ times per week
Sweets	❑	❑ →	☐☐ times per week
Peanuts/ peanut butter	❑	❑ →	☐☐ times per week
Tree nuts/ tree nut butter (i.e. almond, cashew)	❑	❑ →	☐☐ times per week

6. Please indicate if your baby ***currently*** has any of the following feeding behaviours:

	**No**	**Yes**	
Slow feeding	❑	❑	
Taking only small quantities at each feed	❑	❑	
Choking	❑	❑	
Hungry/ not satisfied	❑	❑	
Refused to take breast milk	❑	❑	❑ **Not applicable**
Refused to take other milk	❑	❑	❑ **Not applicable**
Refused to take solids	❑	❑	❑ **Not applicable**
Can not establish feeding routine	❑	❑	

7. Are you ***currently*** having difficulties feeding your baby?
   - ❑ No, no difficulties
   - ❑ Yes, some difficulties
   - ❑ Yes, great difficulties

4 MONTHS

---

8. ***Since your last study visit***, has ***your baby*** had an infection that required antibiotic or antifungal (anti-yeast) treatment?
   - ❑ No
   - ❑ Yes→ if yes, please complete the following table:

DRUG 1

What was the type of infection? ❑ Thrush ❑ Ear infection ❑ Chest infection ❑ Urinary tract infection ❑ Other: ____________________

What was the name of the drug? ____

How was it given? ❑ Topical ❑ Oral ❑ Other: ______________

What was the start date? [ ][ ][ ][ ] / [ ][ ] / [ ][ ] (year / month / day)

What was the end date? [ ][ ][ ][ ] / [ ][ ] / [ ][ ] (year / month / day) or ❑ Still currently in use

---

DRUG 2 ❑ Only one drug was given. (Do not complete the rest of the table)

What was the type of infection? ❑ Thrush ❑ Ear infection ❑ Chest infection ❑ Urinary tract infection ❑ Other: ____________________

What was the name of the second drug? ____

How was it given? ❑ Topical ❑ Oral ❑ Other: ______________

What was the start date? [ ][ ][ ][ ] / [ ][ ] / [ ][ ] (year / month / day)

What was the end date? [ ][ ][ ][ ] / [ ][ ] / [ ][ ] (year / month / day) or ❑ Still currently in use

---

9. Please list any other medications, vitamins, homeopathics or supplements that ***your baby*** has taken ***since your last study visit***.

☐ None

Medication, vitamin or supplement name	Currently in Use? No	Currently in Use? Yes
a. ____	☐	☐
b. ____	☐	☐
c. ____	☐	☐
d. ____	☐	☐
e. ____	☐	☐

10. ***Since your last study visit***, has your baby needed to stay in the hospital?
    - ☐ No
    - ☐ Yes

Baby & Mi

PID: [ ][ ] – [ ][ ][ ]

Mother's Date of Birth: [ ][ ][ ][ ] [ ][ ] [ ][ ]
year month day

4 MONTHS

---

11. ***Since your last study visit***, have ***you*** taken any antibiotic or antifungal (anti-yeast) medications?
    - ❑ No
    - ❑ Yes→ if yes, please complete the following table:

DRUG 1

What was the name of the drug? ______________________________

How was it given? ❑ Topical ❑ Oral ❑ Other: ____________

What was the start date? [ ][ ][ ][ ] / [ ][ ] / [ ][ ]
year month day

What was the end date? [ ][ ][ ][ ] / [ ][ ] / [ ][ ] or ❑ Still currently in use
year month day

DRUG 2 ❑ Only one drug was taken. (Do not complete the rest of the table)

What was the name of the second drug? ______________________________

How was it given? ❑ Topical ❑ Oral ❑ Other: ____________

What was the start date? [ ][ ][ ][ ] / [ ][ ] / [ ][ ]
year month day

What was the end date? [ ][ ][ ][ ] / [ ][ ] / [ ][ ] or ❑ Still currently in use
year month day

---

4 MONTHS

12. ***If you are currently breastfeeding***, please list any other prescription medications that ***you*** have taken ***since your last study visit***.

    ☐ Not currently breastfeeding
    ☐ None

Medication name	Currently in Use? No	Currently in Use? Yes
a. ________________	☐	☐
b. ________________	☐	☐
c. ________________	☐	☐
d. ________________	☐	☐
e. ________________	☐	☐

13. How much time does your baby spend in sleep during the night (between 7 in the evening and 7 in the morning)? ☐☐ hours, ☐☐ minutes

14. How many times does your baby wake up during the night?

    ☐☐ times

15. How much time during the night does your baby spend in wakefulness (from 10 in the evening to 6 in the morning)? ☐☐ hours, ☐☐ minutes

16. Do you consider your baby's sleep to be a problem?
    - ☐ Not a problem at all
    - ☐ A small problem
    - ☐ A very serious problem

17. Has your baby been diagnosed with asthma or reactive airway disease by your doctor?
    - ☐ Yes
    - ☐ No

Baby & Mi

PID: [ ][ ] – [ ][ ][ ]

Mother's Date of Birth: [ ][ ][ ][ ] year [ ][ ] month [ ][ ] day

4 MONTHS

---

18. Does your baby take any puffers or breathing medications?
    - ❑ Yes
    - ❑ No

19. Have you been told by your doctor that your baby has eczema?
    - ❑ Yes
    - ❑ No

20. ***In the last month***, has your baby experienced any of the following?: (check all that apply)
    - ❑ Itchy rashes
    - ❑ Itchy rashes that don't go away on the face, knuckles, elbows or knees
    - ❑ Rash on the nose, mouth or diaper area
    - ❑ Dry, thickened or scaly skin or more skin creases in the palms than usual
    - ❑ Cracked skin around the ear
    - ❑ Scaly scalp that won't go away
    - ❑ Red dots surrounding hair follicles
    - ❑ None of the above

21. Have you ***ever*** had any concerns that your baby may have an allergy?
    - ❑ No
    - ❑ Yes, I/we suspect(ed) an allergy
      - ↳ if yes, please describe what the allergic substance was thought to be and what made you suspect and allergy:

        ____

        ____

        ____

    - ❑ Yes, an allergy was confirmed by the doctor
      - ↳ if yes, please indicate what the allergic substance(s) is/are:

        ____

        ____

        ____

PID: __ __ – __ __ __

Mother's Date of Birth: __ __ __ __ (year) __ __ (month) __ __ (day)

5 MONTHS – PART 1

Instructions:

- **This form is to be completed by research personnel at the 5 month study visit.**
- **Record the measurements indicated below and attach this form to "Part 2" completed by the participant.**

1. Date and time of visit: __ __ __ __ / __ __ / __ __ (year / month / day) __ __ : __ __ (24 hour clock)

2. Head circumference: __ __ . __ cm

3. Tricep skinfold thickness:

   Measurement 1: __ __ . __ cm

   Measurement 2: __ __ . __ cm

   Measurement 3: __ __ . __ cm

4. Subscapular skinfold thickness:

   Measurement 1: __ __ . __ cm

   Measurement 2: __ __ . __ cm

   Measurement 3: __ __ . __ cm

5. Bicep skinfold thickness:

   Measurement 1: __ __ . __ cm

   Measurement 2: __ __ . __ cm

   Measurement 3: __ __ . __ cm

5 MONTHS – PART 1

6. Mid-arm circumference:

   Measurement 1: __ __ . __ cm

   Measurement 2: __ __ . __ cm

   Measurement 3: __ __ . __ cm

7. Suprailiac skinfold thickness:

   Measurement 1: __ __ . __ cm

   Measurement 2: __ __ . __ cm

   Measurement 3: __ __ . __ cm

8. Hip circumference:

   Measurement 1: __ __ . __ cm

   Measurement 2: __ __ . __ cm

   Measurement 3: __ __ . __ cm

9. Abdominal circumference:

   Measurement 1: __ __ . __ cm

   Measurement 2: __ __ . __ cm

   Measurement 3: __ __ . __ cm

10. Length: __ __ __ . __ cm

Baby & Mi

PID: ☐☐ – ☐☐☐

Mother's Date of Birth: ☐☐☐☐ ☐☐ ☐☐
year month day

5 MONTHS – PART 1

---

11. Percent fat: ☐☐ . ☐ %
12. Percent fat free mass: ☐☐ . ☐ %
13. Fat mass: ☐☐ . ☐☐☐☐ kg
14. Fat free mass: ☐☐ . ☐☐☐☐ kg
15. Body mass: ☐☐ . ☐☐☐☐ kg
16. Body volume: ☐☐ . ☐☐☐☐ L
17. Body density: ☐☐ . ☐☐☐☐ kg/L
18. Fat mass density: ☐☐ . ☐☐☐☐ kg/L
19. Fat free mass density: ☐☐ . ☐☐☐☐ kg/L
20. Body surface area: ☐☐☐☐ . ☐ cm^2
21. Thoracic gas volume: ☐☐ . ☐☐☐☐ L
22. The PEA POD test was:
    - ☐ Completed without interruption
    - ☐ Begun but terminated early
    - ☐ Not attempted because baby exceeded capacity
    - ☐ Not attempted because parent declined
23. Were stool samples received?
    - ☐ No
    - ☐ Yes
      ↳ if yes, indicate the following:

Date Collected

	year	month	day
**Day 3**	☐☐☐☐ /	☐☐ /	☐☐
**Day 10**	☐☐☐☐ /	☐☐ /	☐☐
**6 Weeks**	☐☐☐☐ /	☐☐ /	☐☐
**12 Weeks**	☐☐☐☐ /	☐☐ /	☐☐
**5 Months**	☐☐☐☐ /	☐☐ /	☐☐

PID: [ ][ ] – [ ][ ][ ]

Mother's Date of Birth: [ ][ ][ ][ ] [ ][ ] [ ][ ]
year month day

5 MONTHS – PART 2

Instructions:
- **Mark only one box with an "X" for each question, unless instructed to do otherwise.**
- **Please use your study calendar to help you remember the information asked for.**
- **All of your answers will remain confidential.**
- **If you do not understand a question, please ask a research staff member.**

1. Have you ***ever*** breast fed your baby?
   - ☐ No
   - ☐ Yes
     - ↳ if yes, ***do you still*** breastfeed your baby?
       - ☐ No
         - ↳ if no, how old was your baby when you stopped breast feeding?

           [ ][ ] . [ ] months

           (fill in "0" if your baby was less than 1 month old)
       - ☐ Yes
         - ↳ if yes, has your baby ***ever*** had anything other than breast milk or water?
           - ☐ No
           - ☐ Yes
             - ↳ if yes, how old was your baby when he or she first had anything other than breast milk or water?

               [ ][ ] . [ ] months

               (fill in "0" if your baby was less than 1 month old)

2. Has your baby ***ever*** had any of the following?

	No	Yes	Current Use
Formula	☐	☐ →	[ ][ ] times per week
Soy milk	☐	☐ →	[ ][ ] times per week
Goat's milk	☐	☐ →	[ ][ ] times per week
Hypo-allergenic formula	☐	☐ →	[ ][ ] times per week
Cow's milk	☐	☐ →	[ ][ ] times per week
Another type of milk or formula: ______________________	☐	☐ →	[ ][ ] times per week

Baby & Mi

PID: ☐☐–☐☐☐

Mother's Date of Birth: ☐☐☐☐ ☐☐ ☐☐
year month day

5 MONTHS – PART 2

---

3. Has your baby ***ever*** had any of the following foods or beverages?

	No	Yes	Current Use
Rice cereal	❑	❑ →	☐☐ times per week
Other cereal	❑	❑ →	☐☐ times per week
Bread or toast	❑	❑ →	☐☐ times per week
Baby cookies	❑	❑ →	☐☐ times per week
Baked goods	❑	❑ →	☐☐ times per week
Dairy products	❑	❑ →	☐☐ times per week
Egg yolk	❑	❑ →	☐☐ times per week
Egg white	❑	❑ →	☐☐ times per week
Meat	❑	❑ →	☐☐ times per week
Fish	❑	❑ →	☐☐ times per week
Raw vegetables	❑	❑ →	☐☐ times per week
Potato	❑	❑ →	☐☐ times per week
Cooked vegetables	❑	❑ →	☐☐ times per week
Legumes	❑	❑ →	☐☐ times per week
Soy products (i.e. tofu)	❑	❑ →	☐☐ times per week
Raw fruit	❑	❑ →	☐☐ times per week
Cooked fruit	❑	❑ →	☐☐ times per week

Baby & Mi

PID: ☐☐ – ☐☐☐

Mother's Date of Birth: ☐☐☐☐ ☐☐ ☐☐
year month day

5 MONTHS – PART 2

	No	Yes	Current Use
Pop	❑	❑ →	☐☐ times per week
Other fizzy drinks	❑	❑ →	☐☐ times per week
Apple juice	❑	❑ →	☐☐ times per week
Other fruit drinks	❑	❑ →	☐☐ times per week
Herbal drink	❑	❑ →	☐☐ times per week
Unpasteurized milk	❑	❑ →	☐☐ times per week
Gripe water	❑	❑ →	☐☐ times per week
Tea (herbal)	❑	❑ →	☐☐ times per week
Tea (caffeinated)	❑	❑ →	☐☐ times per week
Coffee	❑	❑ →	☐☐ times per week
A little alcohol	❑	❑ →	☐☐ times per week
Potato chips	❑	❑ →	☐☐ times per week
Other salty snacks	❑	❑ →	☐☐ times per week
Chocolate	❑	❑ →	☐☐ times per week
Sweets	❑	❑ →	☐☐ times per week
Peanuts/ peanut butter	❑	❑ →	☐☐ times per week
Tree nuts/ tree nut butter (i.e. almond, cashew)	❑	❑ →	☐☐ times per week

PID: [ ][ ] – [ ][ ][ ]

Mother's Date of Birth: [ ][ ][ ][ ] [ ][ ] [ ][ ]
year month day

5 MONTHS – PART 2

---

4. Please indicate if your baby ***currently*** has any of the following feeding behaviours:

	**No**	**Yes**	
Slow feeding	❑	❑	
Taking only small quantities at each feed	❑	❑	
Choking	❑	❑	
Hungry/ not satisfied	❑	❑	
Refused to take breast milk	❑	❑	❑ **Not applicable**
Refused to take other milk	❑	❑	❑ **Not applicable**
Refused to take solids	❑	❑	❑ **Not applicable**
Can not establish feeding routine	❑	❑	

5. Are you ***currently*** having difficulties feeding your baby?
   - ❑ No, no difficulties
   - ❑ Yes, some difficulties
   - ❑ Yes, great difficulties

6. ***During the past week***, how often did your baby usually spit-up (anything coming out of the mouth) during a 24-hour period?
   - ❑ Less than once
   - ❑ 1 to 3 times
   - ❑ 4 to 6 times
   - ❑ More than 6 times

7. ***During the past week***, how much did your baby usually spit-up (anything coming out of the mouth) during a typical episode?
   - ❑ Did not spit up
   - ❑ Less than 1 tablespoonful
   - ❑ 1 tablespoonful to 2 ounces
   - ❑ More than 2 ounces to half the feeding
   - ❑ More than half the feeding

5 MONTHS – PART 2

8. ***During the past week***, how often did spitting up (anything coming out of the mouth) seem to be uncomfortable for your baby, for example, crying, fussing, irritability, etc?
   - ❑ Never
   - ❑ Rarely
   - ❑ Sometimes
   - ❑ Often
   - ❑ Always

9. ***During the past week***, how often did your baby refuse a feeding even when hungry?
   - ❑ Never
   - ❑ Rarely
   - ❑ Sometimes
   - ❑ Often
   - ❑ Always

10. ***During the past week***, how often did your baby stop eating soon after starting even when hungry?
    - ❑ Never
    - ❑ Rarely
    - ❑ Sometimes
    - ❑ Often
    - ❑ Always

11. ***During the past week***, did your baby cry a lot during or within 1 hour after feedings?
    - ❑ Never
    - ❑ Rarely
    - ❑ Sometimes
    - ❑ Often
    - ❑ Always

12. ***During the past week***, did your baby cry or fuss more than usual?
    - ❑ Never
    - ❑ Rarely
    - ❑ Sometimes
    - ❑ Often
    - ❑ Always

5 MONTHS – PART 2

13. ***During the past week***, on average how long did your baby cry or fuss during a 24 hour period?
    - ❑ Less than 10 minutes
    - ❑ 10 minutes to 1 hour
    - ❑ More than 1 hour but less than 3 hours
    - ❑ 3 or more hours

14. ***During the past week***, how often did your baby have hiccups?
    - ❑ Never
    - ❑ Rarely
    - ❑ Sometimes
    - ❑ Often
    - ❑ Always

15. ***During the past week***, how often did your baby have episodes of arching back?
    - ❑ Never
    - ❑ Rarely
    - ❑ Sometimes
    - ❑ Often
    - ❑ Always

16. ***During the past week***, has your baby stopped breathing while awake or struggled to breathe?
    - ❑ No
    - ❑ Yes

17. ***During the past week***, has your baby turned blue or purple?
    - ❑ No
    - ❑ Yes

5 MONTHS – PART 2

18. ***In the last month***, have you noted your baby straining for 10 minutes or longer before successful passage of stool?
    - ❑ No
    - ❑ Yes
      - ↳ if yes, how many times per week?

        ☐☐ times

19. Does your baby typically have ***at least one*** bowel movement every day?
    - ❑ No
      - ↳ if no, how many bowel movements does your baby have in a ***typical week***?

        ☐☐ bowel movements
    - ❑ Yes
      - ↳ if yes, how many bowel movements does your baby have in a ***typical day***?

        ☐☐ bowel movements

20. ***In the last month***, has your baby had any episodes of abdominal pain or discomfort?
    - ❑ No
    - ❑ Yes

21. ***Since your last study visit or phone call***, has your baby needed to stay in the hospital?
    - ❑ No
    - ❑ Yes

Baby & Mi

PID: ☐☐ – ☐☐☐

Mother's Date of Birth: ☐☐☐☐ ☐☐ ☐☐
year month day

5 MONTHS – PART 2

---

22. ***Since your last study visit or phone call***, has ***your baby*** had an infection that required antibiotic or antifungal (anti-yeast) treatment?
    - ❑ No
    - ❑ Yes→ if yes, please complete the following table:

DRUG 1

What was the type of infection? ❑ Thrush ❑ Ear infection ❑ Chest infection ❑ Urinary tract infection ❑ Other: ____________________________

What was the name of the drug? ____

How was it given? ❑ Topical ❑ Oral ❑ Other: ______________________

What was the start date? ☐☐☐☐ / ☐☐ / ☐☐
year month day

What was the end date? ☐☐☐☐ / ☐☐ / ☐☐ or ❑ Still currently in use
year month day

DRUG 2 ❑ Only one drug was given. (Do not complete the rest of the table)

What was the type of infection? ❑ Thrush ❑ Ear infection ❑ Chest infection ❑ Urinary tract infection ❑ Other: ____________________________

What was the name of the second drug? ____

How was it given? ❑ Topical ❑ Oral ❑ Other: ______________________

What was the start date? ☐☐☐☐ / ☐☐ / ☐☐
year month day

What was the end date? ☐☐☐☐ / ☐☐ / ☐☐ or ❑ Still currently in use
year month day

---

5 MONTHS – PART 2

23. Please list any other medications, vitamins, homeopathics or supplements that ***your baby*** has taken ***since your last study visit or phone call***.

❑ None

Medication, vitamin or supplement name	Currently in Use? No	Currently in Use? Yes
a. ________________________	❑	❑
b. ________________________	❑	❑
c. ________________________	❑	❑
d. ________________________	❑	❑
e. ________________________	❑	❑

Baby & Mi

PID: ☐☐ – ☐☐☐

Mother's Date of Birth: ☐☐☐☐ ☐☐ ☐☐
year month day

5 MONTHS – PART 2

---

24. ***Since your last study visit or phone call***, have ***you*** taken any antibiotic or antifungal (anti-yeast) medications?
    - ❑ No
    - ❑ Yes→ if yes, please complete the following table:

DRUG 1

What was the name of the drug? ______________________________

How was it given? ❑ Topical ❑ Oral ❑ Other: ____________________

What was the start date? ☐☐☐☐ / ☐☐ / ☐☐
year month day

What was the end date? ☐☐☐☐ / ☐☐ / ☐☐ or ❑ Still currently in use
year month day

DRUG 2 ❑ Only one drug was taken. (Do not complete the rest of the table)

What was the name of the second drug? ______________________________

How was it given? ❑ Topical ❑ Oral ❑ Other: ____________________

What was the start date? ☐☐☐☐ / ☐☐ / ☐☐
year month day

What was the end date? ☐☐☐☐ / ☐☐ / ☐☐ or ❑ Still currently in use
year month day

---

5 MONTHS – PART 2

25. ***If you are currently breastfeeding***, please list any other prescription medications that ***you*** have taken ***since your last study visit or phone call***.

    ❑ Not currently breastfeeding
    ❑ None

Medication name	Currently in Use? No	Currently in Use? Yes
a. ______________________	❑	❑
b. ______________________	❑	❑
c. ______________________	❑	❑
d. ______________________	❑	❑
e. ______________________	❑	❑

26. How much time does your baby spend in sleep during the night (between 7 in the evening and 7 in the morning)? ☐☐ hours, ☐☐ minutes

27. How many times does your baby wake up during the night?

    ☐☐ times

28. How much time during the night does your baby spend in wakefulness (from 10 in the evening to 6 in the morning)? ☐☐ hours, ☐☐ minutes

29. Do you consider your baby's sleep to be a problem?
    ❑ Not a problem at all
    ❑ A small problem
    ❑ A very serious problem

30. Has your baby been diagnosed with asthma or reactive airway disease by your doctor?
    ❑ Yes
    ❑ No

5 MONTHS – PART 2

31. Does your baby take any puffers or breathing medications?
    - ☐ Yes
    - ☐ No

32. Have you been told by your doctor that your baby has eczema?
    - ☐ Yes
    - ☐ No

33. ***In the last month***, Has your baby experienced any of the following?: (check all that apply)
    - ☐ Itchy rashes
    - ☐ Itchy rashes that don't go away on the face, knuckles, elbows or knees
    - ☐ Rash on the nose, mouth or diaper area
    - ☐ Dry, thickened or scaly skin or more skin creases in the palms than usual
    - ☐ Cracked skin around the ear
    - ☐ Scaly scalp that won't go away
    - ☐ Red dots surrounding hair follicles
    - ☐ None of the above

34. Have you ***ever*** had any concerns that your baby may have an allergy?
    - ☐ No
    - ☐ Yes, I/we suspect(ed) an allergy
      - ↳ if yes, please describe what the allergic substance was thought to be and what made you suspect and allergy:

        ____

        ____

        ____

    - ☐ Yes, an allergy was confirmed by the doctor
      - ↳ if yes, please indicate what the allergic substance(s) is/are:

        ____

        ____

        ____

Baby & Mi

PID: ☐☐ – ☐☐☐

Mother's Date of Birth: ☐☐☐☐ ☐☐ ☐☐
year month day

Month of contact: ❑6 ❑8 ❑10

6, 8 AND 10 MONTHS - TELEPHONE

Instructions:
- **This form is to be completed by research personnel through telephone contact with participants at 6, 8 and 10 months postpartum.**
- **Please refer to previous forms to appropriately prompt and ensure consistency for questions 3-5, 9, 12 and 21.**

1. Was the follow up contact completed?
   - ❑ No
   - ❑ Yes

2. Date: ☐☐☐☐ / ☐☐ / ☐☐
   year month day

3. Have you ***ever*** breast fed your baby?
   - ❑ No
   - ❑ Yes
     - ↳ if yes, ***do you still*** breastfeed your baby?
       - ❑ No
         - ↳ if no, how old was your baby when you stopped breast feeding?

           ☐☐ . ☐ months

           (fill in "0" if your baby was less than 1 month old)
       - ❑ Yes
         - ↳ if yes, has your baby ***ever*** had anything other than breast milk or water?
           - ❑ No
           - ❑ Yes
             - ↳ if yes, how old was your baby when he or she first had anything other than breast milk or water?

               ☐☐ . ☐ months

               (fill in "0" if your baby was less than 1 month old)

4. Has your baby ***ever*** had any of the following?

	No	Yes	Current Use
Formula	❑	❑ →	☐☐ times per week
Soy milk	❑	❑ →	☐☐ times per week
Goat's milk	❑	❑ →	☐☐ times per week
Hypo-allergenic formula	❑	❑ →	☐☐ times per week
Cow's milk	❑	❑ →	☐☐ times per week
Another type of milk or formula: ________________________________	❑	❑ →	☐☐ times per week

Baby & Mi

PID: ☐☐ – ☐☐☐

Mother's Date of Birth: ☐☐☐☐ ☐☐ ☐☐
year month day

Month of contact: ❑6 ❑8 ❑10

6, 8 AND 10 MONTHS - TELEPHONE

---

5. Has your baby ***ever*** had any of the following foods or beverages?

	No	Yes	Current Use
Rice cereal	❑	❑ →	☐☐ times per week
Other cereal	❑	❑ →	☐☐ times per week
Bread or toast	❑	❑ →	☐☐ times per week
Baby cookies	❑	❑ →	☐☐ times per week
Baked goods	❑	❑ →	☐☐ times per week
Dairy products	❑	❑ →	☐☐ times per week
Egg yolk	❑	❑ →	☐☐ times per week
Egg white	❑	❑ →	☐☐ times per week
Meat	❑	❑ →	☐☐ times per week
Fish	❑	❑ →	☐☐ times per week
Raw vegetables	❑	❑ →	☐☐ times per week
Potato	❑	❑ →	☐☐ times per week
Cooked vegetables	❑	❑ →	☐☐ times per week
Legumes	❑	❑ →	☐☐ times per week
Soy products (i.e. tofu)	❑	❑ →	☐☐ times per week
Raw fruit	❑	❑ →	☐☐ times per week
Cooked fruit	❑	❑ →	☐☐ times per week

Baby & Mi

PID: ☐☐ – ☐☐☐

Mother's Date of Birth: ☐☐☐☐ ☐☐ ☐☐
year month day

Month of contact: ❑6 ❑8 ❑10

6, 8 AND 10 MONTHS - TELEPHONE

	No	Yes	Current Use
Pop	❑	❑ →	☐☐ times per week
Other fizzy drinks	❑	❑ →	☐☐ times per week
Apple juice	❑	❑ →	☐☐ times per week
Other fruit drinks	❑	❑ →	☐☐ times per week
Herbal drink	❑	❑ →	☐☐ times per week
Unpasteurized milk	❑	❑ →	☐☐ times per week
Gripe water	❑	❑ →	☐☐ times per week
Tea (herbal)	❑	❑ →	☐☐ times per week
Tea (caffeinated)	❑	❑ →	☐☐ times per week
Coffee	❑	❑ →	☐☐ times per week
A little alcohol	❑	❑ →	☐☐ times per week
Potato chips	❑	❑ →	☐☐ times per week
Other salty snacks	❑	❑ →	☐☐ times per week
Chocolate	❑	❑ →	☐☐ times per week
Sweets	❑	❑ →	☐☐ times per week
Peanuts/ peanut butter	❑	❑ →	☐☐ times per week
Tree nuts/ tree nut butter (i.e. almond, cashew)	❑	❑ →	☐☐ times per week

6. Please indicate if your baby ***currently*** has any of the following feeding behaviours:

	No	Yes	
Slow feeding	❑	❑	
Taking only small quantities at each feed	❑	❑	
Choking	❑	❑	
Hungry/ not satisfied	❑	❑	
Refused to take breast milk	❑	❑	❑ **Not applicable**
Refused to take other milk	❑	❑	❑ **Not applicable**
Refused to take solids	❑	❑	❑ **Not applicable**
Can not establish feeding routine	❑	❑	

7. Are you ***currently*** having difficulties feeding your baby?
   - ❑ No, no difficulties
   - ❑ Yes, some difficulties
   - ❑ Yes, great difficulties

Baby & Mi

PID: ☐☐ – ☐☐☐

Mother's Date of Birth: ☐☐☐☐ ☐☐ ☐☐
year month day

Month of contact: ❑6 ❑8 ❑10

6, 8 AND 10 MONTHS - TELEPHONE

---

8. ***Since your last study visit or phone call***, has ***your baby*** had an infection that required antibiotic or antifungal (anti-yeast) treatment?
   - ❑ No
   - ❑ Yes→if yes, please complete the following table:

DRUG 1

What was the type of infection? ❑ Thrush ❑ Ear infection ❑ Chest infection ❑ Urinary tract infection ❑ Other: ____________________________

What was the name of the drug? ____

How was it given? ❑ Topical ❑ Oral ❑ Other: ____________________

What was the start date? ☐☐☐☐ / ☐☐ / ☐☐
year month day

What was the end date? ☐☐☐☐ / ☐☐ / ☐☐ or ❑ Still currently in use
year month day

DRUG 2 ❑ Only one drug was given. (Do not complete the rest of the table)

What was the type of infection? ❑ Thrush ❑ Ear infection ❑ Chest infection ❑ Urinary tract infection ❑ Other: ____________________________

What was the name of the second drug? ____

How was it given? ❑ Topical ❑ Oral ❑ Other: ____________________

What was the start date? ☐☐☐☐ / ☐☐ / ☐☐
year month day

What was the end date? ☐☐☐☐ / ☐☐ / ☐☐ or ❑ Still currently in use
year month day

---

9. Please list any other medications, vitamins, homeopathics or supplements that ***your baby*** has taken ***since your last study visit or phone call***.

❑ None

Medication, vitamin or supplement name	Currently in Use? No	Currently in Use? Yes
a. ____	❑	❑
b. ____	❑	❑
c. ____	❑	❑
d. ____	❑	❑
e. ____	❑	❑

10. ***Since your last study visit or phone call***, has your baby needed to stay in the hospital?

❑ No
❑ Yes

Baby & Mi

PID: ☐☐ – ☐☐☐

Mother's Date of Birth: ☐☐☐☐ ☐☐ ☐☐
year month day

Month of contact: ☐6 ☐8 ☐10

6, 8 AND 10 MONTHS - TELEPHONE

---

11. ***Since your last study visit or phone call***, have ***you*** taken any antibiotic or antifungal (anti-yeast) medications?
    - ☐ No
    - ☐ Yes→ if yes, please complete the following table:

DRUG 1

What was the name of the drug? ____________________

How was it given? ☐ Topical ☐ Oral ☐ Other: ____________

What was the start date? ☐☐☐☐ / ☐☐ / ☐☐
year month day

What was the end date? ☐☐☐☐ / ☐☐ / ☐☐ or ☐ Still currently in use
year month day

DRUG 2 ☐ Only one drug was taken. (Do not complete the rest of the table)

What was the name of the second drug? ____________________

How was it given? ☐ Topical ☐ Oral ☐ Other: ____________

What was the start date? ☐☐☐☐ / ☐☐ / ☐☐
year month day

What was the end date? ☐☐☐☐ / ☐☐ / ☐☐ or ☐ Still currently in use
year month day

6, 8 AND 10 MONTHS - TELEPHONE

---

12. If you are currently breastfeeding, please list any other prescription medications that ***you*** have taken ***since your last study visit or phone call***.

    ❑ Not currently breastfeeding
    ❑ None

Medication name	Currently in Use? No	Yes
a. ______________________________	❑	❑
b. ______________________________	❑	❑
c. ______________________________	❑	❑
d. ______________________________	❑	❑
e. ______________________________	❑	❑

13. How much time does your baby spend in sleep during the night (between 7 in the evening and 7 in the morning)? ☐☐ hours, ☐☐ minutes

14. How many times does your baby wake up during the night?
    ☐☐ times

15. How much time during the night does your baby spend in wakefulness (from 10 in the evening to 6 in the morning)? ☐☐ hours, ☐☐ minutes

16. Do you consider your baby's sleep to be a problem?
    ❑ Not a problem at all
    ❑ A small problem
    ❑ A very serious problem

17. Has your baby been diagnosed with asthma or reactive airway disease by your doctor?
    ❑ Yes
    ❑ No

PID: ☐☐ – ☐☐☐

Mother's Date of Birth: ☐☐☐☐ ☐☐ ☐☐
year month day

Month of contact: ❑6 ❑8 ❑10

6, 8 AND 10 MONTHS - TELEPHONE

---

18. Does your baby take any puffers or breathing medications?
    - ❑ Yes
    - ❑ No

19. Have you been told by your doctor that your baby has eczema?
    - ❑ Yes
    - ❑ No

20. ***In the last month***, has your baby experienced any of the following?: (check all that apply)
    - ❑ Itchy rashes
    - ❑ Itchy rashes that don't go away on the face, knuckles, elbows or knees
    - ❑ Rash on the nose, mouth or diaper area
    - ❑ Dry, thickened or scaly skin or more skin creases in the palms than usual
    - ❑ Cracked skin around the ear
    - ❑ Scaly scalp that won't go away
    - ❑ Red dots surrounding hair follicles
    - ❑ None of the above

21. Have you ***ever*** had any concerns that your baby may have an allergy?
    - ❑ No
    - ❑ Yes, I/we suspect(ed) an allergy

      ↳ if yes, please describe what the allergic substance was thought to be and what made you suspect and allergy:

      ____

      ____

      ____

    - ❑ Yes, an allergy was confirmed by the doctor

      ↳ if yes, please indicate what the allergic substance(s) is/are:

      ____

      ____

      ____

Baby & Mi

PID: ☐☐ – ☐☐☐

Mother's Date of Birth: ☐☐☐☐ ☐☐ ☐☐
year month day

1 YEAR – PART 1

Instructions:
- **This form is to be completed by research personnel at the 1 year study visit.**
- **Record the measurements indicated below and attach this form to "Part 2" completed by the participant.**

1. Date and time of visit: ☐☐☐☐ / ☐☐ / ☐☐ ☐☐ : ☐☐
   year month day 24 hour clock

2. Head circumference: ☐☐ . ☐ cm

3. Tricep skinfold thickness:

   Measurement 1: ☐☐ . ☐ cm

   Measurement 2: ☐☐ . ☐ cm

   Measurement 3: ☐☐ . ☐ cm

4. Subscapular skinfold thickness:

   Measurement 1: ☐☐ . ☐ cm

   Measurement 2: ☐☐ . ☐ cm

   Measurement 3: ☐☐ . ☐ cm

5. Bicep skinfold thickness:

   Measurement 1: ☐☐ . ☐ cm

   Measurement 2: ☐☐ . ☐ cm

   Measurement 3: ☐☐ . ☐ cm

<u>1 YEAR – PART 1</u>

---

6. Mid-arm circumference:

   Measurement 1: ☐☐ . ☐ cm

   Measurement 2: ☐☐ . ☐ cm

   Measurement 3: ☐☐ . ☐ cm

7. Suprailiac skinfold thickness:

   Measurement 1: ☐☐ . ☐ cm

   Measurement 2: ☐☐ . ☐ cm

   Measurement 3: ☐☐ . ☐ cm

8. Hip circumference:

   Measurement 1: ☐☐ . ☐ cm

   Measurement 2: ☐☐ . ☐ cm

   Measurement 3: ☐☐ . ☐ cm

9. Abdominal circumference:

   Measurement 1: ☐☐ . ☐ cm

   Measurement 2: ☐☐ . ☐ cm

   Measurement 3: ☐☐ . ☐ cm

10. Length: ☐☐☐ . ☐ cm

11. Body mass: ☐☐ . ☐ kg

1 YEAR – PART 1

---

12. Were stool samples received?
    - ❑ No
    - ❑ Yes
      - ↳ if yes, indicate the following:

	Date Collected (year / month / day)
**Day 3**	____ / __ / __
**Day 10**	____ / __ / __
**6 Weeks**	____ / __ / __
**12 Weeks**	____ / __ / __
**5 Months**	____ / __ / __
**1 Year**	____ / __ / __

Baby & Mi

PID: ☐☐ – ☐☐☐

Mother's Date of Birth: ☐☐☐☐ ☐☐ ☐☐
year month day

1 YEAR – PART 2

Instructions:
- **Mark only one box with an "X" for each question, unless instructed to do otherwise.**
- **Please use your study diary to help you remember the information asked for.**
- **All of your answers will remain confidential.**
- **If you do not understand a question, please ask a research staff member.**

1. Have you *ever* breast fed your baby?
   - ☐ No
   - ☐ Yes
     - ↳ if yes, ***do you still*** breastfeed your baby?
       - ☐ No
         - ↳ if no, how old was your baby when you stopped breast feeding?

           ☐☐ . ☐ months

           (fill in "0" if your baby was less than 1 month old)
       - ☐ Yes
         - ↳ if yes, has your baby ***ever*** had anything other than breast milk or water?
           - ☐ No
           - ☐ Yes
             - ↳ if yes, how old was your baby when she or he first had anything other than breast milk or water?

               ☐☐ . ☐ months

               (fill in "0" if your baby was less than 1 month old)

2. Has your baby ***ever*** had any of the following?

	**No**	**Yes**	**Current Use**
Formula	☐	☐ →	☐☐ times per week
Soy milk	☐	☐ →	☐☐ times per week
Goat's milk	☐	☐ →	☐☐ times per week
Hypo-allergenic formula	☐	☐ →	☐☐ times per week
Cow's milk	☐	☐ →	☐☐ times per week
Another type of milk or formula: ______________________	☐	☐ →	☐☐ times per week

Baby & Mi

PID: [ ][ ] – [ ][ ][ ]

Mother's Date of Birth: [ ][ ][ ][ ] year [ ][ ] month [ ][ ] day

1 YEAR – PART 2

---

3. Has your baby ***ever*** had any of the following foods or beverages?

	No	Yes	Current Use
Rice cereal	❑	❑ →	[ ][ ] times per week
Other cereal	❑	❑ →	[ ][ ] times per week
Bread or toast	❑	❑ →	[ ][ ] times per week
Baby cookies	❑	❑ →	[ ][ ] times per week
Baked goods	❑	❑ →	[ ][ ] times per week
Dairy products	❑	❑ →	[ ][ ] times per week
Egg yolk	❑	❑ →	[ ][ ] times per week
Egg white	❑	❑ →	[ ][ ] times per week
Meat	❑	❑ →	[ ][ ] times per week
Fish	❑	❑ →	[ ][ ] times per week
Raw vegetables	❑	❑ →	[ ][ ] times per week
Potato	❑	❑ →	[ ][ ] times per week
Cooked vegetables	❑	❑ →	[ ][ ] times per week
Legumes	❑	❑ →	[ ][ ] times per week
Soy products (i.e. tofu)	❑	❑ →	[ ][ ] times per week
Raw fruit	❑	❑ →	[ ][ ] times per week
Cooked fruit	❑	❑ →	[ ][ ] times per week

Baby & Mi

PID: __ __ – __ __ __

Mother's Date of Birth: __ __ __ __ (year) __ __ (month) __ __ (day)

1 YEAR – PART 2

	No	Yes	Current Use
Pop	❑	❑ →	☐☐ times per week
Other fizzy drinks	❑	❑ →	☐☐ times per week
Apple juice	❑	❑ →	☐☐ times per week
Other fruit drinks	❑	❑ →	☐☐ times per week
Herbal drink	❑	❑ →	☐☐ times per week
Unpasteurized milk	❑	❑ →	☐☐ times per week
Gripe water	❑	❑ →	☐☐ times per week
Tea (herbal)	❑	❑ →	☐☐ times per week
Tea (caffeinated)	❑	❑ →	☐☐ times per week
Coffee	❑	❑ →	☐☐ times per week
A little alcohol	❑	❑ →	☐☐ times per week
Potato chips	❑	❑ →	☐☐ times per week
Other salty snacks	❑	❑ →	☐☐ times per week
Chocolate	❑	❑ →	☐☐ times per week
Sweets	❑	❑ →	☐☐ times per week
Peanuts/ peanut butter	❑	❑ →	☐☐ times per week
Tree nuts/ tree nut butter (i.e. almond, cashew)	❑	❑ →	☐☐ times per week

---

4. Please indicate if your baby ***currently*** has any of the following feeding behaviours:

	**No**	**Yes**	
Slow feeding	❑	❑	
Taking only small quantities at each feed	❑	❑	
Choking	❑	❑	
Hungry/ not satisfied	❑	❑	
Refused to take breast milk	❑	❑	❑ **Not applicable**
Refused to take other milk	❑	❑	❑ **Not applicable**
Refused to take solids	❑	❑	❑ **Not applicable**
Can not establish feeding routine	❑	❑	

5. Are you ***currently*** having difficulties feeding your baby?
   - ❑ No, no difficulties
   - ❑ Yes, some difficulties
   - ❑ Yes, great difficulties

6. ***During the past week***, how often did your baby usually spit-up (anything coming out of the mouth) during a 24-hour period?
   - ❑ Less than once
   - ❑ 1 to 3 times
   - ❑ 4 to 6 times
   - ❑ More than 6 times

7. ***During the past week***, how much did your baby usually spit-up (anything coming out of the mouth) during a typical episode?
   - ❑ Did not spit up
   - ❑ Less than 1 tablespoonful
   - ❑ 1 tablespoonful to 2 ounces
   - ❑ More than 2 ounces to half the feeding
   - ❑ More than half the feeding

PID: [ ][ ] – [ ][ ][ ]

Mother's Date of Birth: [ ][ ][ ][ ] [ ][ ] [ ][ ]
year month day

1 YEAR – PART 2

---

8. ***During the past week***, how often did spitting up (anything coming out of the mouth) seem to be uncomfortable for your baby, for example, crying, fussing, irritability, etc?
   - ☐ Never
   - ☐ Rarely
   - ☐ Sometimes
   - ☐ Often
   - ☐ Always

9. ***During the past week***, how often did your baby refuse a feeding even when hungry?
   - ☐ Never
   - ☐ Rarely
   - ☐ Sometimes
   - ☐ Often
   - ☐ Always

10. ***During the past week***, how often did your baby stop eating soon after starting even when hungry?
   - ☐ Never
   - ☐ Rarely
   - ☐ Sometimes
   - ☐ Often
   - ☐ Always

11. ***During the past week***, did your baby cry a lot during or within 1 hour after feedings?
   - ☐ Never
   - ☐ Rarely
   - ☐ Sometimes
   - ☐ Often
   - ☐ Always

12. ***During the past week***, did your baby cry or fuss more than usual?
   - ☐ Never
   - ☐ Rarely
   - ☐ Sometimes
   - ☐ Often
   - ☐ Always

1 YEAR – PART 2

---

13. ***During the past week***, on average how long did your baby cry or fuss during a 24 hour period?
    - ☐ Less than 10 minutes
    - ☐ 10 minutes to 1 hour
    - ☐ More than 1 hour but less than 3 hours
    - ☐ 3 or more hours

14. ***During the past week***, how often did your baby have hiccups?
    - ☐ Never
    - ☐ Rarely
    - ☐ Sometimes
    - ☐ Often
    - ☐ Always

15. ***During the past week***, how often did your baby have episodes of arching back?
    - ☐ Never
    - ☐ Rarely
    - ☐ Sometimes
    - ☐ Often
    - ☐ Always

16. ***During the past week***, has your baby stopped breathing while awake or struggled to breathe?
    - ☐ No
    - ☐ Yes

17. ***During the past week***, has your baby turned blue or purple?
    - ☐ No
    - ☐ Yes

18. ***In the last month***, have you noted your baby straining for 10 minutes or longer before successful passage of stool?
   - ❑ No
   - ❑ Yes
     - ↳ if yes, how many times per week?
       ☐☐ times

19. Does your baby typically have ***at least one*** bowel movement every day?
   - ❑ No
     - ↳ if no, how many bowel movements does your baby have in a ***typical week***?
       ☐☐ bowel movements
   - ❑ Yes
     - ↳ if yes, how many bowel movements does your baby have in a ***typical day***?
       ☐☐ bowel movements

20. ***In the last month***, has your baby had any episodes of abdominal pain or discomfort?
   - ❑ No
   - ❑ Yes

Baby & Mi

PID: ☐☐ – ☐☐☐

Mother's Date of Birth: ☐☐☐☐ ☐☐ ☐☐
year month day

1 YEAR – PART 2

---

21. ***Since your last study visit or phone call***, has ***your baby*** had an infection that required antibiotic or antifungal (anti-yeast) treatment?
    - ❑ No
    - ❑ Yes→if yes, please complete the following table:

DRUG 1

What was the type of infection? ❑ Thrush ❑ Ear infection ❑ Chest infection
❑ Urinary tract infection ❑ Other: ___________________________

What was the name of the drug? ____

How was it given? ❑ Topical ❑ Oral ❑ Other: ____________________

What was the start date? ☐☐☐☐ / ☐☐ / ☐☐
year month day

What was the end date? ☐☐☐☐ / ☐☐ / ☐☐ or ❑ Still currently in use
year month day

DRUG 2 ❑ Only one drug was given. (Do not complete the rest of the table)

What was the type of infection? ❑ Thrush ❑ Ear infection ❑ Chest infection
❑ Urinary tract infection ❑ Other: ___________________________

What was the name of the second drug? ____

How was it given? ❑ Topical ❑ Oral ❑ Other: ____________________

What was the start date? ☐☐☐☐ / ☐☐ / ☐☐
year month day

What was the end date? ☐☐☐☐ / ☐☐ / ☐☐ or ❑ Still currently in use
year month day

---

22. Please list any other medications, vitamins, homeopathics or supplements that ***your baby*** has taken ***since your last study visit or phone call***.

    ❑ None

Medication, vitamin or supplement name	Currently in Use? No	Currently in Use? Yes
a. ________________________________	❑	❑
b. ________________________________	❑	❑
c. ________________________________	❑	❑
d. ________________________________	❑	❑
e. ________________________________	❑	❑

23. ***Since your last study visit or phone call***, has your baby needed to stay in the hospital?
    ❑ No
    ❑ Yes

PID: ☐☐–☐☐☐

Mother's Date of Birth: ☐☐☐☐ ☐☐ ☐☐
year month day

1 YEAR – PART 2

---

24. ***Since your last study visit or phone call***, have ***you*** taken any antibiotic or antifungal (anti-yeast) medications?
    - ❑ No
    - ❑ Yes→ if yes, please complete the following table:

DRUG 1

What was the name of the drug? ____________________

How was it given? ❑ Topical ❑ Oral ❑ Other: ____________________

What was the start date? ☐☐☐☐ / ☐☐ / ☐☐
year month day

What was the end date? ☐☐☐☐ / ☐☐ / ☐☐ or ❑ Still currently in use
year month day

DRUG 2 ❑ Only one drug was taken. (Do not complete the rest of the table)

What was the name of the second drug? ____________________

How was it given? ❑ Topical ❑ Oral ❑ Other: ____________________

What was the start date? ☐☐☐☐ / ☐☐ / ☐☐
year month day

What was the end date? ☐☐☐☐ / ☐☐ / ☐☐ or ❑ Still currently in use
year month day

---

1 YEAR – PART 2

---

25. ***If you are currently breastfeeding***, please list any other prescription medications that ***you*** have taken ***since your last study visit or phone call***.

    ❑ Not currently breastfeeding
    ❑ None

Medication name	Currently in Use? No	Yes
a. ________________________	❑	❑
b. ________________________	❑	❑
c. ________________________	❑	❑
d. ________________________	❑	❑
e. ________________________	❑	❑

26. How much time does your baby spend in sleep during the night (between 7 in the evening and 7 in the morning)? ☐☐ hours, ☐☐ minutes

27. How many times does your baby wake up during the night?
    ☐☐ times

28. How much time during the night does your baby spend in wakefulness (from 10 in the evening to 6 in the morning)? ☐☐ hours, ☐☐ minutes

29. Do you consider your baby's sleep to be a problem?
    ❑ Not a problem at all
    ❑ A small problem
    ❑ A very serious problem

1 YEAR – PART 2

30. Has your baby been diagnosed with asthma or reactive airway disease by your doctor?
    - ☐ Yes
    - ☐ No

31. Has your baby ***ever*** had wheezing?
    - ☐ Yes
    - ☐ No

32. Has your baby ***ever*** had a problem with sneezing or a runny nose or a blocked nose when he/she **did not** have a cold or flu?
    - ☐ Yes
    - ☐ No

33. Does your baby take any puffers or breathing medications?
    - ☐ Yes
    - ☐ No

34. Have you been told by your doctor that your baby has "hay fever"?
    - ☐ Yes
    - ☐ No

35. Have you been told by your doctor that your baby has eczema?
    - ☐ Yes
    - ☐ No

36. ***In the last month***, has your baby experienced any of the following?: (check all that apply)
    - ☐ Itchy rashes
    - ☐ Itchy rashes that don't go away on the face, knuckles, elbows or knees
    - ☐ Rash on the nose, mouth or diaper area
    - ☐ Dry, thickened or scaly skin or more skin creases in the palms than usual
    - ☐ Cracked skin around the ear
    - ☐ Scaly scalp that won't go away
    - ☐ Red dots surrounding hair follicles
    - ☐ None of the above

---

37. Have you ***ever*** had any concerns that your baby may have an allergy?
    - ☐ No
    - ☐ Yes, I/we suspect(ed) an allergy
      ↳ if yes, please describe what the allergic substance was thought to be and what made you suspect and allergy:

      ____

      ____

      ____

    - ☐ Yes, an allergy was confirmed by the doctor
      ↳ if yes, please indicate what the allergic substance(s) is/are:

      ____

      ____

      ____

38. ***Since your baby was born***, has he/she been regularly exposed to tobacco smoke? (regularly is defined as most days or nights)
    - ☐ No
    - ☐ Yes

39. Has your baby ***ever*** travelled outside of Canada or the United States?
    - ❑ No
    - ❑ Yes → if yes, please indicate the country, date and duration of stay of each trip:

Country	Date of arrival (year / month / day)	Duration of visit
a. ______________	☐☐☐☐ / ☐☐ / ☐☐	______________
b. ______________	☐☐☐☐ / ☐☐ / ☐☐	______________
c. ______________	☐☐☐☐ / ☐☐ / ☐☐	______________
d. ______________	☐☐☐☐ / ☐☐ / ☐☐	______________
e. ______________	☐☐☐☐ / ☐☐ / ☐☐	______________

40. What type of water does your child drink most often?
    - ❑ Well water
    - ❑ Bottled water
    - ❑ Municipal water
    - ❑ Municipal water with home filtration system

PID: __ __ – __ __ __

Mother's Date of Birth: __ __ __ __ (year) __ __ (month) __ __ (day)

1 YEAR – PART 2

---

41. Does your child attend daycare or preschool?
    - ☐ No
      - → if no, who cares for your child? (check all that apply)
        - ☐ Parent
        - ☐ Grandparent / other extended family member
        - ☐ Nanny / babysitter
    - ☐ Yes
      - → if yes, how old was your child when they first attended?
        - __ __ . __ months
      - → if yes, please indicate the type of care:
        - ☐ Home daycare
        - ☐ Centre-based daycare
        - ☐ Preschool
      - → if yes, please indicate the frequency of attendance:
        - ☐ Part-time
        - ☐ Full-time

Baby & Mi

PID: ☐☐ – ☐☐☐

Mother's Date of Birth: ☐☐☐☐ ☐☐ ☐☐
year month day

1.5 YEARS

Instructions:

- **This form is to be completed by research personnel through telephone contact with participants when their child 1.5 years old.**
- **Please refer to previous forms to appropriately prompt and ensure consistency for questions 3, 5, 6, 8, 9 and 14-21.**

1. Was the follow up contact completed?
   - ☐ No
   - ☐ Yes

2. Date: ☐☐☐☐ / ☐☐ / ☐☐
   year month day

3. Have you ***ever*** breast fed your child?
   - ☐ No
   - ☐ Yes
     - ↳ if yes, ***do you still*** breastfeed your child?
       - ☐ No
         - ↳ if no, how old was your child when you stopped breast feeding?

           ☐☐ . ☐ months

           (fill in "0" if your child was less than 1 month old)
       - ☐ Yes

4. Does your child eat a special diet?
   - ☐ No
   - ☐ Yes
     - ↳ if yes, check all that apply:

Vegetarian	☐
Vegan	☐
Gluten free	☐
Dairy free	☐
Other:	☐

____

Baby & Mi

PID: ☐☐ – ☐☐☐

Mother's Date of Birth: ☐☐☐☐ ☐☐ ☐☐
year month day

1.5 YEARS

---

5. ***Since your last study visit or phone call***, has ***your child*** had an infection that required antibiotic or antifungal (anti-yeast) treatment?
   - ❑ No
   - ❑ Yes→if yes, please complete the following table:

DRUG 1

What was the type of infection? ❑ Thrush ❑ Ear infection ❑ Chest infection ❑ Urinary tract infection ❑ Other: ___________________________

What was the name of the drug? ____

How was it given? ❑ Topical ❑ Oral ❑ Other: ____________________

What was the start date? ☐☐☐☐ / ☐☐ / ☐☐
year month day

What was the end date? ☐☐☐☐ / ☐☐ / ☐☐ or ❑ Still currently in use
year month day

DRUG 2 ❑ N/A (Do not complete the rest of the table)

What was the type of infection? ❑ Thrush ❑ Ear infection ❑ Chest infection ❑ Urinary tract infection ❑ Other: ___________________________

What was the name of the second drug? ____

How was it given? ❑ Topical ❑ Oral ❑ Other: ____________________

What was the start date? ☐☐☐☐ / ☐☐ / ☐☐
year month day

What was the end date? ☐☐☐☐ / ☐☐ / ☐☐ or ❑ Still currently in use
year month day

1.5 YEARS

---

6. Please list any other medications, vitamins, homeopathics or supplements that ***your child*** has taken ***since your last study visit or phone call***.

   ❑ None

Medication, vitamin or supplement name	Currently in Use? No	Yes
a. ____	❑	❑
b. ____	❑	❑
c. ____	❑	❑
d. ____	❑	❑
e. ____	❑	❑
f. ____	❑	❑
g. ____	❑	❑

7. ***Since your last study visit or phone call***, has your child needed to stay in the hospital?
   ❑ No
   ❑ Yes

Baby & Mi

PID: ☐☐ – ☐☐☐

Mother's Date of Birth: ☐☐☐☐ ☐☐ ☐☐
year month day

1.5 YEARS

---

8. ***If you are currently breastfeeding,*** have ***you*** taken any antibiotic or antifungal (anti-yeast) medications ***since your last study visit or phone call***?
   - ☐ Not currently breastfeeding
   - ☐ No
   - ☐ Yes→ if yes, please complete the following table:

DRUG 1

What was the name of the drug? ______________________________

How was it given? ☐ Topical ☐ Oral ☐ Other: ____________

What was the start date? ☐☐☐☐ / ☐☐ / ☐☐
year month day

What was the end date? ☐☐☐☐ / ☐☐ / ☐☐ or ☐ Still currently in use
year month day

DRUG 2 ☐ Only one drug was taken. (Do not complete the rest of the table)

What was the name of the second drug? ______________________________

How was it given? ☐ Topical ☐ Oral ☐ Other: ____________

What was the start date? ☐☐☐☐ / ☐☐ / ☐☐
year month day

What was the end date? ☐☐☐☐ / ☐☐ / ☐☐ or ☐ Still currently in use
year month day

---

9. ***If you are currently breastfeeding***, please list any other prescription medications that ***you*** have taken ***since your last study visit or phone call***.

   ❑ Not currently breastfeeding
   ❑ None

Medication name	Currently in Use? No	Currently in Use? Yes
a. ____________________	❑	❑
b. ____________________	❑	❑
c. ____________________	❑	❑
d. ____________________	❑	❑
e. ____________________	❑	❑
f. ____________________	❑	❑
g. ____________________	❑	❑

10. How much time does your child spend in sleep during the night (between 7 in the evening and 7 in the morning)? ☐☐ hours, ☐☐ minutes

11. How many times does your child wake up during the night?
    ☐☐ times

12. How much time during the night does your child spend in wakefulness (from 10 in the evening to 6 in the morning)? ☐☐ hours, ☐☐ minutes

13. Do you consider your child's sleep to be a problem?
    ❑ Not a problem at all
    ❑ A small problem
    ❑ A very serious problem

1.5 YEARS

---

14. Has your child been diagnosed with asthma or reactive airway disease by your doctor?
    - ❑ Yes
    - ❑ No

15. Has your child ***ever*** had wheezing?
    - ❑ Yes
    - ❑ No

16. Has your child ***ever*** had a problem with sneezing or a runny nose or a blocked nose when he/she **did not** have a cold or flu?
    - ❑ Yes
    - ❑ No

17. Does your child take any puffers or breathing medications?
    - ❑ Yes
    - ❑ No

18. Have you been told by your doctor that your child has "hay fever"?
    - ❑ Yes
    - ❑ No

19. Have you been told by your doctor that your child has eczema?
    - ❑ Yes
    - ❑ No

20. Has your child ***ever*** had an itchy rash which was coming and going for at least 6 months?
    - ❑ No
    - ❑ Yes

      ↳ if yes, has this itchy rash at any time affected any of the following places: the folds of the elbows, behind the knees, in front of the ankles, under the buttocks, or around the neck ears or eyes?
        - ❑ Yes
        - ❑ No

21. Have you ***ever*** been told by your doctor that your child has an allergy?
    - ❑ No
    - ❑ Yes

      ↳ if yes, please specify the allergic substance(s) :

      ____

      ____

Baby & Mi

PID: ☐☐ – ☐☐☐

Mother's Date of Birth: ☐☐☐☐ year ☐☐ month ☐☐ day

2 YEARS – PART 1

Instructions:
- **This form is to be completed by research personnel at the 2 year study visit.**
- **Record the measurements indicated below and attach this form to "Part 2" completed by the participant.**

1. Date and time of visit: ☐☐☐☐ / ☐☐ / ☐☐ ☐☐ : ☐☐
   year month day 24 hour clock

2. Head circumference: ☐☐ . ☐ cm

3. Tricep skinfold thickness:

   Measurement 1: ☐☐ . ☐ cm

   Measurement 2: ☐☐ . ☐ cm

   Measurement 3: ☐☐ . ☐ cm

4. Subscapular skinfold thickness:

   Measurement 1: ☐☐ . ☐ cm

   Measurement 2: ☐☐ . ☐ cm

   Measurement 3: ☐☐ . ☐ cm

5. Bicep skinfold thickness:

   Measurement 1: ☐☐ . ☐ cm

   Measurement 2: ☐☐ . ☐ cm

   Measurement 3: ☐☐ . ☐ cm

2 YEARS – PART 1

6. Mid-arm circumference:

   Measurement 1: ☐☐ . ☐ cm

   Measurement 2: ☐☐ . ☐ cm

   Measurement 3: ☐☐ . ☐ cm

7. Suprailiac skinfold thickness:

   Measurement 1: ☐☐ . ☐ cm

   Measurement 2: ☐☐ . ☐ cm

   Measurement 3: ☐☐ . ☐ cm

8. Hip circumference:

   Measurement 1: ☐☐ . ☐ cm

   Measurement 2: ☐☐ . ☐ cm

   Measurement 3: ☐☐ . ☐ cm

9. Abdominal circumference:

   Measurement 1: ☐☐ . ☐ cm

   Measurement 2: ☐☐ . ☐ cm

   Measurement 3: ☐☐ . ☐ cm

10. Length: ☐☐☐ . ☐ cm

11. Body mass: ☐☐ . ☐ kg

Baby & Mi

PID: ☐☐ – ☐☐☐

Mother's Date of Birth: ☐☐☐☐ ☐☐ ☐☐
year month day

2 YEARS – PART 1

---

12. Were stool samples received?
    - ❑ No
    - ❑ Yes
      - ↳ if yes, indicate the following:

	Date Collected
**Day 3**	☐☐☐☐ / ☐☐ / ☐☐ (year / month / day)
**Day 10**	☐☐☐☐ / ☐☐ / ☐☐
**6 Weeks**	☐☐☐☐ / ☐☐ / ☐☐
**12 Weeks**	☐☐☐☐ / ☐☐ / ☐☐
**5 Months**	☐☐☐☐ / ☐☐ / ☐☐
**1 Year**	☐☐☐☐ / ☐☐ / ☐☐
**2 Year**	☐☐☐☐ / ☐☐ / ☐☐

Baby & Mi

PID: ☐☐ – ☐☐☐

Mother's Date of Birth: ☐☐☐☐ ☐☐ ☐☐
year month day

2 YEARS – PART 2

Instructions:

- **Mark only one box with an "X" for each question, unless instructed to do otherwise.**
- **Please use your study diary to help you remember the information asked for.**
- **All of your answers will remain confidential.**
- **If you do not understand a question, please ask a research staff member.**

1. Have you ***ever*** breast fed your child?
   - ☐ No
   - ☐ Yes
     - ↳ if yes, ***do you still*** breastfeed your child?
       - ☐ No
         - ↳ if no, how old was your child when you stopped breast feeding?
           ☐☐ . ☐ months
           (fill in "0" if your child was less than 1 month old)
       - ☐ Yes

2. Does your child eat a special diet?
   - ☐ No
   - ☐ Yes
     - ↳ if yes, check all that apply:

Vegetarian	☐
Vegan	☐
Gluten free	☐
Dairy free	☐
Other: ______________________________	☐

2 YEARS – PART 2

---

3. Are you ***currently*** having difficulties feeding your child?
   - ☐ No, no difficulties
   - ☐ Yes, some difficulties
   - ☐ Yes, great difficulties

4. Does your child typically have ***at least one*** bowel movement every day?
   - ☐ No
     - ↳ if no, how many bowel movements does your child have in a ***typical week***?
       ☐☐ bowel movements
   - ☐ Yes
     - ↳ if yes, how many bowel movements does your child have in a ***typical day***?
       ☐☐ bowel movements

5. ***In the last month***, has your child had any episodes of abdominal pain or discomfort?
   - ☐ No
   - ☐ Yes

Baby & Mi

PID: ☐☐–☐☐☐

Mother's Date of Birth: ☐☐☐☐ ☐☐ ☐☐
year month day

2 YEARS – PART 2

---

6. ***Since your last study visit or phone call***, has ***your child*** had an infection that required antibiotic or antifungal (anti-yeast) treatment?
   - ❑ No
   - ❑ Yes→if yes, please complete the following table:

DRUG 1

What was the type of infection? ❑ Thrush ❑ Ear infection ❑ Chest infection ❑ Urinary tract infection ❑ Other: ____________________________

What was the name of the drug? ____

How was it given? ❑ Topical ❑ Oral ❑ Other: ______________________

What was the start date? ☐☐☐☐ / ☐☐ / ☐☐
year month day

What was the end date? ☐☐☐☐ / ☐☐ / ☐☐ or ❑ Still currently in use
year month day

DRUG 2 ❑ N/A (Do not complete the rest of the table)

What was the type of infection? ❑ Thrush ❑ Ear infection ❑ Chest infection ❑ Urinary tract infection ❑ Other: ____________________________

What was the name of the second drug? ____

How was it given? ❑ Topical ❑ Oral ❑ Other: ______________________

What was the start date? ☐☐☐☐ / ☐☐ / ☐☐
year month day

What was the end date? ☐☐☐☐ / ☐☐ / ☐☐ or ❑ Still currently in use
year month day

---

7. Please list any other medications, vitamins, homeopathics or supplements that ***your child*** has taken ***since your last study visit or phone call***.

   ☐ None

Medication, vitamin or supplement name	Currently in Use? No	Currently in Use? Yes
a. ______________________	☐	☐
b. ______________________	☐	☐
c. ______________________	☐	☐
d. ______________________	☐	☐
e. ______________________	☐	☐
f. ______________________	☐	☐
g. ______________________	☐	☐

8. ***Since your last study visit or phone call***, has your child needed to stay in the hospital?
   - ☐ No
   - ☐ Yes

Baby & Mi

PID: ☐☐ – ☐☐☐

Mother's Date of Birth: ☐☐☐☐ ☐☐ ☐☐
year month day

2 YEARS – PART 2

---

9. ***If you are currently breastfeeding,*** have ***you*** taken any antibiotic or antifungal (anti-yeast) medications ***since your last study visit or phone call***?
   - ☐ Not currently breastfeeding
   - ☐ No
   - ☐ Yes→ if yes, please complete the following table:

DRUG 1

What was the name of the drug? ____________________

How was it given? ☐ Topical ☐ Oral ☐ Other: ____________________

What was the start date? ☐☐☐☐ / ☐☐ / ☐☐
year month day

What was the end date? ☐☐☐☐ / ☐☐ / ☐☐ or ☐ Still currently in use
year month day

DRUG 2 ☐ Only one drug was taken. (Do not complete the rest of the table)

What was the name of the second drug? ____________________

How was it given? ☐ Topical ☐ Oral ☐ Other: ____________________

What was the start date? ☐☐☐☐ / ☐☐ / ☐☐
year month day

What was the end date? ☐☐☐☐ / ☐☐ / ☐☐ or ☐ Still currently in use
year month day

---

2 YEARS – PART 2

---

10. ***If you are currently breastfeeding***, please list any other prescription medications that ***you*** have taken ***since your last study visit or phone call***.

- ☐ Not currently breastfeeding
- ☐ None

Medication name	Currently in Use? No	Currently in Use? Yes
a. ________________	☐	☐
b. ________________	☐	☐
c. ________________	☐	☐
d. ________________	☐	☐
e. ________________	☐	☐
f. ________________	☐	☐
g. ________________	☐	☐

11. How much time does your child spend in sleep during the night (between 7 in the evening and 7 in the morning)? ☐☐ hours, ☐☐ minutes

12. How many times does your child wake up during the night?
☐☐ times

13. How much time during the night does your child spend in wakefulness (from 10 in the evening to 6 in the morning)? ☐☐ hours, ☐☐ minutes

14. Do you consider your child's sleep to be a problem?
- ☐ Not a problem at all
- ☐ A small problem
- ☐ A very serious problem

2 YEARS – PART 2

---

15. Has your child been diagnosed with asthma or reactive airway disease by your doctor?
    - ❑ Yes
    - ❑ No

16. Has your child ***ever*** had wheezing?
    - ❑ Yes
    - ❑ No

17. Has your child ***ever*** had a problem with sneezing or a runny nose or a blocked nose when he/she **did not** have a cold or flu?
    - ❑ Yes
    - ❑ No

18. Does your child take any puffers or breathing medications?
    - ❑ Yes
    - ❑ No

19. Have you been told by your doctor that your child has "hay fever"?
    - ❑ Yes
    - ❑ No

20. Have you been told by your doctor that your child has eczema?
    - ❑ Yes
    - ❑ No

21. Has your child ***ever*** had an itchy rash which was coming and going for at least 6 months?
    - ❑ No
    - ❑ Yes
      - ↳ if yes, has this itchy rash at any time affected any of the following places: the folds of the elbows, behind the knees, in front of the ankles, under the buttocks, or around the neck ears or eyes?
        - ❑ Yes
        - ❑ No

2 YEARS – PART 2

---

22. Have you ***ever*** been told by your doctor that your child has an allergy?
    - ❑ No
    - ❑ Yes
      ↳ if yes, please specify the allergic substance(s) :

      ____

      ____

23. ***In the last year***, has your child been regularly exposed to tobacco smoke? (regularly is defined as most days or nights)
    - ❑ No
    - ❑ Yes

24. ***In the last year,*** has your child travelled outside of Canada or the United States?
    - ❑ No
    - ❑ Yes → if yes, please indicate the country, date and duration of stay of each trip:

Country	Date of arrival (year / month / day)	Duration of visit
a. ____________	☐☐☐☐ / ☐☐ / ☐☐	____________
b. ____________	☐☐☐☐ / ☐☐ / ☐☐	____________
c. ____________	☐☐☐☐ / ☐☐ / ☐☐	____________
d. ____________	☐☐☐☐ / ☐☐ / ☐☐	____________
e. ____________	☐☐☐☐ / ☐☐ / ☐☐	____________

25. What type of water does your child drink most often?
    - ❑ Well water
    - ❑ Bottled water
    - ❑ Municipal water
    - ❑ Municipal water with home filtration system

---

26. Does your child attend daycare or preschool?
    - ☐ No
      - ↳ if no, who cares for your child? (check all that apply)
        - ☐ Parent
        - ☐ Grandparent / other extended family member
        - ☐ Nanny / babysitter
    - ☐ Yes
      - → if yes, how old was your child when they first attended?

        ☐☐ . ☐ months
      - → if yes, please indicate the type of care:
        - ☐ Home daycare
        - ☐ Centre-based daycare
        - ☐ Preschool
      - → if yes, please indicate the frequency of attendance:
        - ☐ Part-time
        - ☐ Full-time

Baby & Mi

PID: ☐☐ – ☐☐☐

Mother's Date of Birth: ☐☐☐☐ ☐☐ ☐☐
year month day

2.5 YEARS

Instructions:
- **This form is to be completed by research personnel through telephone contact with participants when their child 2.5 years old.**
- **Please refer to previous forms to appropriately prompt and ensure consistency for questions 3, 5, 6, 8, 9 and 14-21.**

1. Was the follow up contact completed?
   - ☐ No
   - ☐ Yes

2. Date: ☐☐☐☐ / ☐☐ / ☐☐
   year month day

3. Have you ***ever*** breast fed your child?
   - ☐ No
   - ☐ Yes
     - ↳ if yes, ***do you still*** breastfeed your child?
       - ☐ No
         - ↳ if no, how old was your child when you stopped breast feeding?
           ☐☐ . ☐ months
           (fill in "0" if your child was less than 1 month old)
       - ☐ Yes

4. Does your child eat a special diet?
   - ☐ No
   - ☐ Yes
     - ↳ if yes, check all that apply:

Vegetarian	☐
Vegan	☐
Gluten free	☐
Dairy free	☐
Other:	☐

____

Baby & Mi

PID: ☐☐ – ☐☐☐

Mother's Date of Birth: ☐☐☐☐ ☐☐ ☐☐
year month day

2.5 YEARS

---

5. ***Since your last study visit or phone call***, has ***your child*** had an infection that required antibiotic or antifungal (anti-yeast) treatment?
   - ☐ No
   - ☐ Yes→if yes, please complete the following table:

DRUG 1

What was the type of infection? ☐ Thrush ☐ Ear infection ☐ Chest infection ☐ Urinary tract infection ☐ Other: ___________________________

What was the name of the drug? ____

How was it given? ☐ Topical ☐ Oral ☐ Other: ____________________

What was the start date? ☐☐☐☐ / ☐☐ / ☐☐
year month day

What was the end date? ☐☐☐☐ / ☐☐ / ☐☐ or ☐ Still currently in use
year month day

DRUG 2 ☐ N/A (Do not complete the rest of the table)

What was the type of infection? ☐ Thrush ☐ Ear infection ☐ Chest infection ☐ Urinary tract infection ☐ Other: ___________________________

What was the name of the second drug? ____

How was it given? ☐ Topical ☐ Oral ☐ Other: ____________________

What was the start date? ☐☐☐☐ / ☐☐ / ☐☐
year month day

What was the end date? ☐☐☐☐ / ☐☐ / ☐☐ or ☐ Still currently in use
year month day

2.5 YEARS

6. Please list any other medications, vitamins, homeopathics or supplements that ***your child*** has taken ***since your last study visit or phone call***.

   ☐ None

Medication, vitamin or supplement name	Currently in Use? No	Currently in Use? Yes
a. ________________________________	☐	☐
b. ________________________________	☐	☐
c. ________________________________	☐	☐
d. ________________________________	☐	☐
e. ________________________________	☐	☐
f. ________________________________	☐	☐
g. ________________________________	☐	☐

7. ***Since your last study visit or phone call***, has your child needed to stay in the hospital?
   ☐ No
   ☐ Yes

Baby & Mi

PID: ☐☐ – ☐☐☐

Mother's Date of Birth: ☐☐☐☐ ☐☐ ☐☐
year month day

2.5 YEARS

---

8. ***If you are currently breastfeeding,*** have ***you*** taken any antibiotic or antifungal (anti-yeast) medications ***since your last study visit or phone call***?
   - ❑ Not currently breastfeeding
   - ❑ No
   - ❑ Yes→ if yes, please complete the following table:

DRUG 1

What was the name of the drug? ______________________________

How was it given? ❑ Topical ❑ Oral ❑ Other: ____________

What was the start date? ☐☐☐☐ / ☐☐ / ☐☐
year month day

What was the end date? ☐☐☐☐ / ☐☐ / ☐☐ or ❑ Still currently in use
year month day

DRUG 2 ❑ Only one drug was taken. (Do not complete the rest of the table)

What was the name of the second drug? ______________________________

How was it given? ❑ Topical ❑ Oral ❑ Other: ____________

What was the start date? ☐☐☐☐ / ☐☐ / ☐☐
year month day

What was the end date? ☐☐☐☐ / ☐☐ / ☐☐ or ❑ Still currently in use
year month day

---

9. ***If you are currently breastfeeding***, please list any other prescription medications that ***you*** have taken ***since your last study visit or phone call***.

   ❑ Not currently breastfeeding
   ❑ None

Medication name	Currently in Use? No	Currently in Use? Yes
a. ________________________________	❑	❑
b. ________________________________	❑	❑
c. ________________________________	❑	❑
d. ________________________________	❑	❑
e. ________________________________	❑	❑
f. ________________________________	❑	❑
g. ________________________________	❑	❑

10. How much time does your child spend in sleep during the night (between 7 in the evening and 7 in the morning)? __ __ hours, __ __ minutes

11. How many times does your child wake up during the night?

    __ __ times

12. How much time during the night does your child spend in wakefulness (from 10 in the evening to 6 in the morning)? __ __ hours, __ __ minutes

13. Do you consider your child's sleep to be a problem?
    ❑ Not a problem at all
    ❑ A small problem
    ❑ A very serious problem

2.5 YEARS

---

14. Has your child been diagnosed with asthma or reactive airway disease by your doctor?
    - ☐ Yes
    - ☐ No

15. Has your child ***ever*** had wheezing?
    - ☐ Yes
    - ☐ No

16. Has your child ***ever*** had a problem with sneezing or a runny nose or a blocked nose when he/she **did not** have a cold or flu?
    - ☐ Yes
    - ☐ No

17. Does your child take any puffers or breathing medications?
    - ☐ Yes
    - ☐ No

18. Have you been told by your doctor that your child has "hay fever"?
    - ☐ Yes
    - ☐ No

19. Have you been told by your doctor that your child has eczema?
    - ☐ Yes
    - ☐ No

20. Has your child ***ever*** had an itchy rash which was coming and going for at least 6 months?
    - ☐ No
    - ☐ Yes
      - ↳ if yes, has this itchy rash at any time affected any of the following places: the folds of the elbows, behind the knees, in front of the ankles, under the buttocks, or around the neck ears or eyes?
        - ☐ Yes
        - ☐ No

21. Have you ***ever*** been told by your doctor that your child has an allergy?
    - ☐ No
    - ☐ Yes
      - ↳ if yes, please specify the allergic substance(s) :

        ____

        ____

PID: ☐☐ – ☐☐☐

Mother's Date of Birth: ☐☐☐☐ ☐☐ ☐☐
year month day

3 YEARS – PART 1

Instructions:

- **This form is to be completed by research personnel at the 3 year study visit.**
- **Record the measurements indicated below and attach this form to "Part 2" completed by the participant.**

1. Date and time of visit: ☐☐☐☐ / ☐☐ / ☐☐ ☐☐ : ☐☐
   year month day 24 hour clock

2. Head circumference: ☐☐ . ☐ cm

3. Tricep skinfold thickness:

   Measurement 1: ☐☐ . ☐ cm

   Measurement 2: ☐☐ . ☐ cm

   Measurement 3: ☐☐ . ☐ cm

4. Subscapular skinfold thickness:

   Measurement 1: ☐☐ . ☐ cm

   Measurement 2: ☐☐ . ☐ cm

   Measurement 3: ☐☐ . ☐ cm

5. Bicep skinfold thickness:

   Measurement 1: ☐☐ . ☐ cm

   Measurement 2: ☐☐ . ☐ cm

   Measurement 3: ☐☐ . ☐ cm

3 YEARS – PART 1

---

6. Mid-arm circumference:

   Measurement 1: [ ][ ] . [ ] cm

   Measurement 2: [ ][ ] . [ ] cm

   Measurement 3: [ ][ ] . [ ] cm

7. Suprailiac skinfold thickness:

   Measurement 1: [ ][ ] . [ ] cm

   Measurement 2: [ ][ ] . [ ] cm

   Measurement 3: [ ][ ] . [ ] cm

8. Hip circumference:

   Measurement 1: [ ][ ] . [ ] cm

   Measurement 2: [ ][ ] . [ ] cm

   Measurement 3: [ ][ ] . [ ] cm

9. Abdominal circumference:

   Measurement 1: [ ][ ] . [ ] cm

   Measurement 2: [ ][ ] . [ ] cm

   Measurement 3: [ ][ ] . [ ] cm

10. Length: [ ][ ][ ] . [ ] cm

11. Body mass: [ ][ ] . [ ] kg

3 YEARS – PART 1

12. Were stool samples received?
    - ❑ No
    - ❑ Yes
      - ↳ if yes, indicate the following:

	Date Collected
**Day 3**	☐☐☐☐ / ☐☐ / ☐☐ (year / month / day)
**Day 10**	☐☐☐☐ / ☐☐ / ☐☐
**6 Weeks**	☐☐☐☐ / ☐☐ / ☐☐
**12 Weeks**	☐☐☐☐ / ☐☐ / ☐☐
**5 Months**	☐☐☐☐ / ☐☐ / ☐☐
**1 Year**	☐☐☐☐ / ☐☐ / ☐☐
**2 Year**	☐☐☐☐ / ☐☐ / ☐☐
**3 Year**	☐☐☐☐ / ☐☐ / ☐☐

13. What is the child's ethnicity?
    - ❑ Asian
    - ❑ Black
    - ❑ Hispanic
    - ❑ White
    - ❑ Other

3 YEARS – PART 1

14. Was a blood sample taken?
    - ☐ No
      - if no, indicate the following:
        - ☐ Not attempted
        - ☐ Attempted, not able to obtain a sample
        - ☐ Parent declined
        - ☐ Child declined
    - ☐ Yes
      - if yes, indicate the following:

        Date Collected

        ☐☐☐☐ / ☐☐ / ☐☐
        year month day

      - if yes, when did your child last eat or drink anything except water?
        - ☐ < 1 hour before
        - ☐ 1-3 hours before
        - ☐ > 3 hours before
        - ☐ Overnight
      - if yes, did your child experience any of the following in the 48 hours prior to blood collection?

	**No**	**Yes**
Fever	☐	☐
Runny/stuffy nose	☐	☐
Cough	☐	☐
Diarrhea or vomiting	☐	☐

3 YEARS – PART 1

15. Was a DXA scan performed?
    - ☐ No
      - ↳ if no, indicate the following:
        - ☐ Not attempted
        - ☐ Attempted, not able to obtain a reading
        - ☐ Parent declined
        - ☐ Child declined
    - ☐ Yes
      - ↳ if yes, indicate the following:

Date Performed

☐☐☐☐ / ☐☐ / ☐☐
year month day

↳ if yes, indicate the following:

a) **Bone** (Ancillary Results [Total Body] page, Summary Sheet)

i. BA ☐☐☐☐☐ . ☐☐ cm^2

ii. BMC ☐☐☐☐☐ . ☐☐ g

iii. BMD ☐☐☐ . ☐ g/cm^2

iv. BMD z-score (age-matched) ☐ (+ / -) ☐ . ☐

b) **Fat and Lean Mass** (Body Composition page)

i. Fat ☐☐☐☐☐ . ☐ g

ii. Lean ☐☐☐☐☐☐ . ☐ g

iii. % Fat ☐☐☐ . ☐ %

iv. Total Mass ☐☐ . ☐ kg

c) **Fat Distribution** – Trunk (Body Composition page)

i. Android (% fat) __ __ . __ %

ii. Gynoid (% fat) __ __ . __ %

iii. Trunk (% fat) __ __ . __ %

iv. Fat (trunk total) __ __ __ __ __ . __ g

v. Lean (trunk total) __ __ __ __ __ . __ g

d) **Fat Mass Ratio** (Body Composition page)

vi. Trunk/Total __ __ . __ __

vii. Legs/Total __ __ . __ __

viii. Arms+Legs/Total __ __ . __ __

PID: ☐☐–☐☐☐

Mother's Date of Birth: ☐☐☐☐ ☐☐ ☐☐
year month day

3 YEARS – PART 2

Instructions:

- **Mark only one box with an "X" for each question, unless instructed to do otherwise.**
- **Please use your study diary to help you remember the information asked for.**
- **All of your answers will remain confidential.**
- **If you do not understand a question, please ask a research staff member.**

1. Have you ***ever*** breast fed your child?
   - ❑ No
   - ❑ Yes
     - ↳ if yes, ***do you still*** breastfeed your child?
       - ❑ No
         - ↳ if no, how old was your child when you stopped breast feeding?

           ☐☐ . ☐ months

           (fill in "0" if your child was less than 1 month old)
       - ❑ Yes

2. Does your child eat a special diet?
   - ❑ No
   - ❑ Yes
     - ↳ if yes, check all that apply:

Vegetarian	❑
Vegan	❑
Gluten free	❑
Dairy free	❑
Other:	❑

____

3 YEARS – PART 2

3. Are you ***currently*** having difficulties feeding your child?
   - ❑ No, no difficulties
   - ❑ Yes, some difficulties
   - ❑ Yes, great difficulties

4. Does your child typically have ***at least one*** bowel movement every day?
   - ❑ No
     ↳ if no, how many bowel movements does your child have in a ***typical week***?
     ☐☐ bowel movements
   - ❑ Yes
     ↳ if yes, how many bowel movements does your child have in a ***typical day***?
     ☐☐ bowel movements

5. ***In the last month***, has your child had any episodes of abdominal pain or discomfort?
   - ❑ No
   - ❑ Yes

<u>3 YEARS – PART 2</u>

Below are questions about your child's eating and other habits. Think about your child's every day habits when answering. Check only one answer for each question.

6. My child usually eats grain products (examples are bread, bagel, bun, cereal, pasta, rice, roti and tortillas):
   - ☐ More than 5 times a day
   - ☐ 4 to 5 times a day
   - ☐ 2 to 3 times a day
   - ☐ Less than 2 times a day

7. My child usually has milk products (examples are white or chocolate milk, cheese, yogurt, milk puddings or milk substitutes, such as fortified soy beverages):
   - ☐ More than 3 times a day
   - ☐ 3 times a day
   - ☐ 2 times a day
   - ☐ Once a day or less

8. My child usually eats fruit:
   - ☐ More than 3 times a day
   - ☐ 3 times a day
   - ☐ 2 times a day
   - ☐ Once a day
   - ☐ Not at all

9. My child usually eats vegetables:
   - ☐ More than 2 times a day
   - ☐ 2 times a day
   - ☐ Once a day
   - ☐ Not at all

10. My child usually eats meat, fish, poultry or alternatives (alternatives can be eggs, peanut butter, tofu, nuts or fried beans, peas and lentils):
    - ☐ More than 2 times a day
    - ☐ 2 times a day
    - ☐ Once a day
    - ☐ A few times a week
    - ☐ Not at all

Baby & Mi

PID: [ ][ ] – [ ][ ][ ]

Mother's Date of Birth: [ ][ ][ ][ ] [ ][ ] [ ][ ]
year month day

3 YEARS – PART 2

---

11. My child usually eats "fast food":
    - ☐ 4 or more times a week
    - ☐ 2 to 3 times a week
    - ☐ Once a week
    - ☐ A few times a month
    - ☐ Once a month or less

12. I have difficulty buying food to feed my child because food is expensive:
    - ☐ Most of the time
    - ☐ Sometimes
    - ☐ Rarely
    - ☐ Never

13. My child has problems chewing, swallowing, gagging or choking when eating:
    - ☐ Most of the time
    - ☐ Sometimes
    - ☐ Rarely
    - ☐ Never

14. My child is not hungry at mealtimes because he/she drinks all day:
    - ☐ Most of the time
    - ☐ Sometimes
    - ☐ Rarely
    - ☐ Never

15. My child usually eats:
    - ☐ Less than 2 times a day
    - ☐ 2 times a day
    - ☐ 3 to 4 times a day
    - ☐ 5 times a day
    - ☐ More than 5 times a day

16. I let my child decide how much to eat:
    - ☐ Always
    - ☐ Most of the time
    - ☐ Sometimes
    - ☐ Rarely
    - ☐ Never

PID: [ ][ ] – [ ][ ][ ]

Mother's Date of Birth: [ ][ ][ ][ ] [ ][ ] [ ][ ]
year month day

3 YEARS – PART 2

---

17. My child eats meals while watching TV:
    - ❑ Always
    - ❑ Most of the time
    - ❑ Sometimes
    - ❑ Rarely
    - ❑ Never

18. My child usually takes supplements: (Examples are multivitamins, iron drops, cod liver oil)
    - ❑ Always
    - ❑ Most of the time
    - ❑ Sometimes
    - ❑ Rarely
    - ❑ Never

19. My child:
    - ❑ Needs more physical activity
    - ❑ Gets enough physical activity

20. My child usually watches TV, uses the computer or plays video games:
    - ❑ 5 or more hours a day
    - ❑ 4 hours a day
    - ❑ 3 hours a day
    - ❑ 2 hours a day
    - ❑ 1 hour or less a day

21. I am comfortable with how my child is growing:
    - ❑ Yes
    - ❑ No

22. My child:
    - ❑ Should weight more
    - ❑ Is about the right weight
    - ❑ Should weigh less

Baby & Mi

PID: ☐☐ – ☐☐☐

Mother's Date of Birth: ☐☐☐☐ ☐☐ ☐☐
year month day

3 YEARS – PART 2

---

23. ***Since your last study visit or phone call***, has ***your child*** had an infection that required antibiotic or antifungal (anti-yeast) treatment?
    - ❑ No
    - ❑ Yes→if yes, please complete the following table:

DRUG 1

What was the type of infection? ❑ Thrush ❑ Ear infection ❑ Chest infection ❑ Urinary tract infection ❑ Other: ___________________________

What was the name of the drug? ____

How was it given? ❑ Topical ❑ Oral ❑ Other: ____________________

What was the start date? ☐☐☐☐ / ☐☐ / ☐☐
year month day

What was the end date? ☐☐☐☐ / ☐☐ / ☐☐ or ❑ Still currently in use
year month day

DRUG 2 ❑ N/A (Do not complete the rest of the table)

What was the type of infection? ❑ Thrush ❑ Ear infection ❑ Chest infection ❑ Urinary tract infection ❑ Other: ___________________________

What was the name of the second drug? ____

How was it given? ❑ Topical ❑ Oral ❑ Other: ____________________

What was the start date? ☐☐☐☐ / ☐☐ / ☐☐
year month day

What was the end date? ☐☐☐☐ / ☐☐ / ☐☐ or ❑ Still currently in use
year month day

---

24. Please list any other medications, vitamins, homeopathics or supplements that ***your child*** has taken ***since your last study visit or phone call***.

☐ None

Medication, vitamin or supplement name	Currently in Use? No	Currently in Use? Yes
a. ______________________	☐	☐
b. ______________________	☐	☐
c. ______________________	☐	☐
d. ______________________	☐	☐
e. ______________________	☐	☐
f. ______________________	☐	☐
g. ______________________	☐	☐

25. ***Since your last study visit or phone call***, has your child needed to stay in the hospital?
    - ☐ No
    - ☐ Yes

3 YEARS – PART 2

---

26. ***If you are currently breastfeeding,*** have ***you*** taken any antibiotic or antifungal (anti-yeast) medications ***since your last study visit or phone call***?
    - ☐ Not currently breastfeeding
    - ☐ No
    - ☐ Yes→ if yes, please complete the following table:

DRUG 1

What was the name of the drug? ____________________

How was it given? ☐ Topical ☐ Oral ☐ Other: ____________________

What was the start date? [ ][ ][ ][ ] / [ ][ ] / [ ][ ]
year month day

What was the end date? [ ][ ][ ][ ] / [ ][ ] / [ ][ ] or ☐ Still currently in use
year month day

DRUG 2 ☐ Only one drug was taken. (Do not complete the rest of the table)

What was the name of the second drug? ____________________

How was it given? ☐ Topical ☐ Oral ☐ Other: ____________________

What was the start date? [ ][ ][ ][ ] / [ ][ ] / [ ][ ]
year month day

What was the end date? [ ][ ][ ][ ] / [ ][ ] / [ ][ ] or ☐ Still currently in use
year month day

---

27. ***If you are currently breastfeeding***, please list any other prescription medications that ***you*** have taken ***since your last study visit or phone call***.

- ❑ Not currently breastfeeding
- ❑ None

Medication name	Currently in Use? No	Currently in Use? Yes
a. ____	❑	❑
b. ____	❑	❑
c. ____	❑	❑
d. ____	❑	❑
e. ____	❑	❑
f. ____	❑	❑
g. ____	❑	❑

28. How much time does your child spend in sleep during the night (between 7 in the evening and 7 in the morning)? [ ][ ] hours, [ ][ ] minutes

29. How many times does your child wake up during the night?

[ ][ ] times

30. How much time during the night does your child spend in wakefulness (from 10 in the evening to 6 in the morning)? [ ][ ] hours, [ ][ ] minutes

31. Do you consider your child's sleep to be a problem?
    - ❑ Not a problem at all
    - ❑ A small problem
    - ❑ A very serious problem

---

32. Has your child been diagnosed with asthma or reactive airway disease by your doctor?
    - ☐ Yes
    - ☐ No

33. Has your child ***ever*** had wheezing?
    - ☐ Yes
    - ☐ No

34. Has your child ***ever*** had a problem with sneezing or a runny nose or a blocked nose when he/she **did not** have a cold or flu?
    - ☐ Yes
    - ☐ No

35. Does your child take any puffers or breathing medications?
    - ☐ Yes
    - ☐ No

36. Have you been told by your doctor that your child has "hay fever"?
    - ☐ Yes
    - ☐ No

Baby & Mi

PID: ☐☐ – ☐☐☐

Mother's Date of Birth: ☐☐☐☐ ☐☐ ☐☐
year month day

37. Has your child ***ever*** had an itchy rash which was coming and going for at least 6 months?
    - ❑ No
    - ❑ Yes
      - → If yes, has your child has this itchy rash at any time in the last 12 months?
        - ❑ No
        - ❑ Yes
          - → If yes, has this itchy rash at any time affected any of the following places: the folds of the elbows, behind the knees, in front of the ankles, under the buttocks, or around the neck ears or eyes?
            - ❑ Yes
            - ❑ No
          - → If yes, at what age did this itchy rash first occur? One year of age or less
            - ❑ Under 1 year
            - ❑ Age 1 to 2
            - ❑ Age 2 or more
          - → If yes, has this rash cleared completely at any time during the last 12 months?
            - ❑ Yes
            - ❑ No
          - → If yes, in the last 12 months, how often, on average, has your child been kept awake at night by this itchy rash?
            - ❑ Never in the last 12 months
            - ❑ Less than one night per week
            - ❑ One or more nights per week

38. Has your child ***ever*** had eczema?
    - ❑ Yes
    - ❑ No

3 YEARS – PART 2

---

39. Have you ***ever*** been told by your doctor that your child has an allergy?
    - ❑ No
    - ❑ Yes
      - ↳ if yes, please specify the allergic substance(s) :

        ____

        ____

40. ***In the last year***, has your child been regularly exposed to tobacco smoke? (regularly is defined as most days or nights)
    - ❑ No
    - ❑ Yes

41. ***In the last year,*** has your child travelled outside of Canada or the United States?
    - ❑ No
    - ❑ Yes → if yes, please indicate the country, date and duration of stay of each trip:

Country	Date of arrival (year / month / day)	Duration of visit
a. ____________	☐☐☐☐ / ☐☐ / ☐☐	____________
b. ____________	☐☐☐☐ / ☐☐ / ☐☐	____________
c. ____________	☐☐☐☐ / ☐☐ / ☐☐	____________
d. ____________	☐☐☐☐ / ☐☐ / ☐☐	____________
e. ____________	☐☐☐☐ / ☐☐ / ☐☐	____________

42. What type of water does your child drink most often?
    - ❑ Well water
    - ❑ Bottled water
    - ❑ Municipal water
    - ❑ Municipal water with home filtration system

PID: ☐☐ – ☐☐☐

Mother's Date of Birth: ☐☐☐☐ ☐☐ ☐☐
year month day

3 YEARS – PART 2

43. Does your child attend daycare or preschool?
    - ☐ No
      - if no, who cares for your child? (check all that apply)
        - ☐ Parent
        - ☐ Grandparent / other extended family member
        - ☐ Nanny / babysitter
    - ☐ Yes
      - if yes, how old was your child when they first attended?

        ☐☐ . ☐ months
      - if yes, please indicate the type of care:
        - ☐ Home daycare
        - ☐ Centre-based daycare
        - ☐ Preschool
      - if yes, please indicate the frequency of attendance:
        - ☐ Part-time
        - ☐ Full-time

44. We are interested in knowing about your child's exposure to household animals at your home or the home of a regular care provider. If your child has been regularly exposed (most days or nights) to animals during any of the age ranges listed below, please indicate it by checking the boxes that apply.

	**Never**	**Less than 3 months old**	**3 months to 6 months old**	**6 months to 1 year old**	**1 year to 2 years old**	**2 years to 3 years old**
Animals that stayed **inside** the house.	☐	☐	☐	☐	☐	☐
Animals that went **outside and inside** the house	☐	☐	☐	☐	☐	☐
Animals that stayed **outside** the house	☐	☐	☐	☐	☐	☐

PID: [ ][ ]–[ ][ ][ ]

Mother's Date of Birth: [ ][ ][ ][ ] [ ][ ] [ ][ ]
year month day

3 YEARS – PART 2

For each of the items below please mark the box for "Not True", "Somewhat True" or "Certainly True" based on ***your child's*** behaviour ***over the last 6 months***. Please answer all items as best as you can even if you are not absolutely certain.

	Not true	Somewhat true	Certainly true
45. Considerate of other people's feelings	❑	❑	❑
46. Restless, overactive, cannot stay still for long	❑	❑	❑
47. Often complains of headaches, stomach-aches or sickness	❑	❑	❑
48. Shares readily with other children, for example toys, treats, pencils	❑	❑	❑
49. Often loses temper	❑	❑	❑
50. Rather solitary, prefers to play alone	❑	❑	❑
51. Generally well behaved, usually does what adults request	❑	❑	❑
52. Many worries or often seems worried	❑	❑	❑
53. Helpful if someone is hurt, upset or feeling ill	❑	❑	❑
54. Constantly fidgeting or squirming	❑	❑	❑
55. Has at least one good friend	❑	❑	❑
56. Often fights with other children or bullies them	❑	❑	❑
57. Often unhappy, depressed or tearful	❑	❑	❑
58. Generally liked by other children	❑	❑	❑
59. Easily distracted, concentration wanders	❑	❑	❑
60. Nervous or clingy in new situations, easily loses confidence	❑	❑	❑
61. Kind to younger children	❑	❑	❑
62. Often argumentative with adults	❑	❑	❑
63. Picked on or bullied by other children	❑	❑	❑
64. Often offers to help others (parents, teachers, other children)	❑	❑	❑
65. Can stop and think things out before acting	❑	❑	❑
66. Can be spiteful to others	❑	❑	❑
67. Gets along better with adults than with other children	❑	❑	❑
68. Many fears, easily scared	❑	❑	❑
69. Good attention span, sees work through to the end	❑	❑	❑

3 YEARS – PART 2

---

70. Overall, do you think that your child has difficulties in one or more of the following areas: emotions, concentration, behaviour, or being able to get along with other people?
    - ☐ No
    - ☐ Yes, minor difficulties
    - ☐ Yes, definite difficulties
    - ☐ Yes, severe difficulties

→ If yes, how long have these difficulties been present?
- ☐ Less than a month
- ☐ 1 to 5 months
- ☐ 6 to 12 months
- ☐ Over a year

→ If yes, do the difficulties upset or distress your child?
- ☐ Not at all
- ☐ Only a little
- ☐ A medium amount
- ☐ A great deal

→ If yes, do the difficulties interfere with your child's everyday life in the following areas?

	**Not at all**	**Only a little**	**A medium amount**	**A great deal**
Home life	☐	☐	☐	☐
Friendships	☐	☐	☐	☐
Learning	☐	☐	☐	☐
Leisure activities	☐	☐	☐	☐

→ If yes, do the difficulties put a burden on you or the family as a whole?
- ☐ Not at all
- ☐ Only a little
- ☐ A medium amount
- ☐ A great deal

3 YEARS – PART 2

On the next pages you will see a set of statements that describe children's reactions to a number of situations. We would like you to tell us what ***your child's*** reaction is likely to be in those situations. There are of course no "correct" ways of reacting; children differ widely in their reactions and it is these differences that we are trying to learn about. Please read each statement and decide whether it is a "true" or "untrue" description of your child's reaction ***within the past six months***. Use the following scale to indicate how well a statement describes your child:

1 extremely untrue of your child

2 quite untrue of your child

3 slightly untrue of your child

4 neither true nor untrue of your child

5 slightly true of your child

6 quite true of your child

7 extremely true of your child

If you cannot answer one of the items because you have never seen the child in that situation, then circle NA (not applicable). Please be sure to circle a number or NA for every item.

3 YEARS – PART 2

1	2	3	4	5	6	7	NA
Extremely untrue	Quite untrue	Slightly untrue	Neither true nor untrue	Slightly true	Quite true	Extremely true	Not applicable

My child:

71. Seems always in a big hurry to get from one place to another.
1 2 3 4 5 6 7 NA

72. Gets quite frustrated when prevented from doing something s/he wants to do.
1 2 3 4 5 6 7 NA

73. When drawing or colouring in a book, shows strong concentration.
1 2 3 4 5 6 7 NA

74. Likes going down high slides or other adventurous activities.
1 2 3 4 5 6 7 NA

75. Is quite upset by a little cut or bruise.
1 2 3 4 5 6 7 NA

76. Prepares for trips and outings by planning things s/he will need.
1 2 3 4 5 6 7 NA

77. Often rushes into new situations.
1 2 3 4 5 6 7 NA

78. Tends to become sad if the family's plans don't work out.
1 2 3 4 5 6 7 NA

79. Likes being sung to.
1 2 3 4 5 6 7 NA

80. Seems to be at ease with almost any person.
1 2 3 4 5 6 7 NA

81. Is afraid of burglars or the "boogie man".
1 2 3 4 5 6 7 NA

Baby & Mi

PID: ☐☐ – ☐☐☐

Mother's Date of Birth: ☐☐☐☐ ☐☐ ☐☐
year month day

3 YEARS – PART 2

1	2	3	4	5	6	7	NA
Extremely untrue	Quite untrue	Slightly untrue	Neither true nor untrue	Slightly true	Quite true	Extremely true	Not applicable

My child:

82. Notices it when parents are wearing new clothing.
    1 2 3 4 5 6 7 NA

83. Prefers quiet activities to active games.
    1 2 3 4 5 6 7 NA

84. When angry about something, s/he tends to stay upset for ten minutes or longer.
    1 2 3 4 5 6 7 NA

85. When building or putting something together, becomes very involved in what s/he is doing and works for long periods.
    1 2 3 4 5 6 7 NA

86. Likes to go high and fast when pushed on a swing.
    1 2 3 4 5 6 7 NA

87. Seems to feel depressed when unable to accomplish some task.
    1 2 3 4 5 6 7 NA

88. Is good at following instructions.
    1 2 3 4 5 6 7 NA

89. Takes a long time in approaching new situations.
    1 2 3 4 5 6 7 NA

90. Hardly ever complains when ill with a cold.
    1 2 3 4 5 6 7 NA

91. Likes the sound of words, such as nursery rhymes.
    1 2 3 4 5 6 7 NA

92. Is sometimes shy even around people s/he has known a long time.
    1 2 3 4 5 6 7 NA

Baby & Mi

PID: [ ][ ] – [ ][ ][ ]

Mother's Date of Birth: [ ][ ][ ][ ] [ ][ ] [ ][ ]
year month day

3 YEARS – PART 2

1	2	3	4	5	6	7	NA
Extremely untrue	Quite untrue	Slightly untrue	Neither true nor untrue	Slightly true	Quite true	Extremely true	Not applicable

My child:

93. Is very difficult to soothe when s/he has become upset.
1 2 3 4 5 6 7 NA

94. Is quickly aware of some new item in the living room.
1 2 3 4 5 6 7 NA

95. Is full of energy, even in the evening.
1 2 3 4 5 6 7 NA

96. Is not afraid of the dark.
1 2 3 4 5 6 7 NA

97. Sometimes becomes absorbed in a picture book and looks at it for a long time.
1 2 3 4 5 6 7 NA

98. Likes rough and rowdy games.
1 2 3 4 5 6 7 NA

99. Is not very upset at minor cuts or bruises.
1 2 3 4 5 6 7 NA

100. Approaches places s/he has been told are dangerous slowly and cautiously.
1 2 3 4 5 6 7 NA

101. Is slow and unhurried in deciding what to do next.
1 2 3 4 5 6 7 NA

102. Gets angry when s/he can't find something s/he wants to play with.
1 2 3 4 5 6 7 NA

103. Enjoys gentle rhythmic activities such as rocking or swaying.
1 2 3 4 5 6 7 NA

Baby & Mi

PID: | | |–| | | |

Mother's Date of Birth: year month day

3 YEARS – PART 2

1	2	3	4	5	6	7	NA
Extremely untrue	Quite untrue	Slightly untrue	Neither true nor false	Slightly true	Quite true	Extremely true	Not applicable

My child:

104. Sometimes turns away shyly from new acquaintances.
1 2 3 4 5 6 7 NA

105. Becomes upset when loved relatives or friends are getting ready to leave following a visit.
1 2 3 4 5 6 7 NA

106. Comments when a parent has changed his/her appearance.
1 2 3 4 5 6 7 NA

Baby & Mi

PID: ☐☐ – ☐☐☐

Mother's Date of Birth: ☐☐☐☐ ☐☐ ☐☐
year month day

3 YEARS – PART 2

---

107. Have you, your child's father, or your other child/children (if applicable) experienced any of the following:

	Yourself			Father of Child			Other child/children ❑ N/A		
	No	Yes	Unknown	No	Yes	Unknown	No	Yes	Unknown
Inflammatory Bowel Disease	❑	❑	❑	❑	❑	❑	❑	❑	❑
Autoimmune Disease	❑	❑	❑	❑	❑	❑	❑	❑	❑
Depression (only since your child was born)	❑	❑	❑	❑	❑	❑	❑	❑	❑

108. What is your annual household income?
- ❑ Less than $25,000
- ❑ $25,000 to $49,999
- ❑ $50,000 to $74,999
- ❑ $75,000 to $99,999
- ❑ $100,000 to $124,999
- ❑ $125,000 or more

109. What is the highest level of education that ***you*** have completed?
- ❑ Elementary school
- ❑ High school / secondary school
- ❑ College diploma
- ❑ Postsecondary apprenticeship or training certificate
- ❑ University undergraduate degree
- ❑ Graduate degree
- ❑ Postgraduate degree

110. If you were to join a similar study in the future, how would you like to be reminded to collect diaper samples?
- ❑ Email
- ❑ Telephone call
- ❑ Cell phone text message
- ❑ By my midwife, doctor or other care provider
- ❑ I wouldn't like to be reminded

Baby & Mi

PID: ☐☐ – ☐☐☐

Mother's Date of Birth: ☐☐☐☐ ☐☐ ☐☐
year month day

3 YEARS – PART 2

---

111. What did you find to be the most challenging throughout your participation in this study?

____

____

____

112. What did you enjoy the most about participating in this study?

____

____

____

113. Would you choose to participate in a similar study, if you were invited to do so in the future?
- ☐ No
- ☐ Yes

Baby & Mi

PID: ☐☐ – ☐☐☐

Mother's Date of Birth: ☐☐☐☐ (year) ☐☐ (month) ☐☐ (day)

VACCINATION RECORD

Vaccination Date YYYY / MM / DD	DTAP - Diptheria, Tetanus, Pertussis	IPV - Injected Polio Vaccine	OPV - Oral Polio Vaccine	HiB - Haemophilus influenzae type B	Pneumococcal	Rotavirus	Meningococcal	MMR - Mumps, Measles, Rubella	Varicella	Influenza	Other #1	Other #2	Other #3	Other #4	Other #5
__ __ __ __/ __ __/ __ __															
__ __ __ __/ __ __/ __ __															
__ __ __ __/ __ __/ __ __															
__ __ __ __/ __ __/ __ __															
__ __ __ __/ __ __/ __ __															
__ __ __ __/ __ __/ __ __															
__ __ __ __/ __ __/ __ __															
__ __ __ __/ __ __/ __ __															
__ __ __ __/ __ __/ __ __															
__ __ __ __/ __ __/ __ __															
__ __ __ __/ __ __/ __ __															
__ __ __ __/ __ __/ __ __															
__ __ __ __/ __ __/ __ __															
__ __ __ __/ __ __/ __ __															
__ __ __ __/ __ __/ __ __															
__ __ __ __/ __ __/ __ __															
